# Benefits of Curcumin in the Vasculature: A Therapeutic Candidate for Vascular Remodeling in Arterial Hypertension and Pulmonary Arterial Hypertension?

**DOI:** 10.3389/fphys.2022.848867

**Published:** 2022-04-01

**Authors:** Ke-Xue Li, Zi-Chao Wang, Jeremiah Ong’Achwa Machuki, Meng-Zhen Li, Yu-Jie Wu, Ming-Kai Niu, Kang-Ying Yu, Qing-Bo Lu, Hai-Jian Sun

**Affiliations:** ^1^ Department of Physiology, Xuzhou Medical University, Xuzhou, China; ^2^ State Key Laboratory of Natural Medicines, China Pharmaceutical University, Nanjing, China; ^3^ School of Traditional Chinese Pharmacy, China Pharmaceutical University, Nanjing, China; ^4^ Nursing School of Wuxi Taihu University, Wuxi, China; ^5^ School of Medicine, Southeast University, Nanjing, China; ^6^ Department of Pharmacology, Yong Loo Lin School of Medicine, National University of Singapore, Singapore, Singapore

**Keywords:** hypertension, vascular remodeling, curcumin, vascular smooth muscle cells, endothelial cells

## Abstract

Growing evidence suggests that hypertension is one of the leading causes of cardiovascular morbidity and mortality since uncontrolled high blood pressure increases the risk of myocardial infarction, aortic dissection, hemorrhagic stroke, and chronic kidney disease. Impaired vascular homeostasis plays a critical role in the development of hypertension-induced vascular remodeling. Abnormal behaviors of vascular cells are not only a pathological hallmark of hypertensive vascular remodeling, but also an important pathological basis for maintaining reduced vascular compliance in hypertension. Targeting vascular remodeling represents a novel therapeutic approach in hypertension and its cardiovascular complications. Phytochemicals are emerging as candidates with therapeutic effects on numerous pathologies, including hypertension. An increasing number of studies have found that curcumin, a polyphenolic compound derived from dietary spice turmeric, holds a broad spectrum of pharmacological actions, such as antiplatelet, anticancer, anti-inflammatory, antioxidant, and antiangiogenic effects. Curcumin has been shown to prevent or treat vascular remodeling in hypertensive rodents by modulating various signaling pathways. In the present review, we attempt to focus on the current findings and molecular mechanisms of curcumin in the treatment of hypertensive vascular remodeling. In particular, adverse and inconsistent effects of curcumin, as well as some favorable pharmacokinetics or pharmacodynamics profiles in arterial hypertension will be discussed. Moreover, the recent progress in the preparation of nano-curcumins and their therapeutic potential in hypertension will be briefly recapped. The future research directions and challenges of curcumin in hypertension-related vascular remodeling are also proposed. It is foreseeable that curcumin is likely to be a therapeutic agent for hypertension and vascular remodeling going forwards.

## Introduction

Arterial hypertension, a multifactorial and chronic disease, is one of the leading causes of people disability and death around the world (Forouzanfar et al., 2017; Carey et al., 2018). As a common type of hypertension, pulmonary arterial hypertension (PAH) is a devastating disorder that is manifested by progressive pulmonary arteriole remodeling, vasoconstriction, pulmonary vascular stiffening, and increased right ventricular afterload (Thenappan et al., 2018). Studies have shown that hypertension originates from a combination of genetic, environmental, and social determinants (Carey et al., 2018). Uncontrolled high blood pressure results in a higher incidence of hypertension-related target organ damages, including myocardial infarction, heart failure, aortic dissection, renal damage, and stroke (Acelajado et al., 2019; Van Beusecum and Moreno, 2021). Large-scale epidemiological studies provide robust evidence that high blood pressure is closely associated with the risk of stroke, ischemic heart disease, heart failure, and non-cardiac vascular disease, without heterogeneity at all ages and in both sexes (Rapsomaniki et al., 2014). In 2019, the American College of Cardiology and American Heart Association classified hypertension as a systolic blood pressure >130 mmHg and a diastolic blood pressure of >80 mmHg (Arnett et al., 2019). These guidelines lead to the fact that nearly half of adults tend to be hypertensive (Arnett et al., 2019). Moreover, the prevalence of PAH ranges from 11 to 26 cases per million adults, and the mortality rate remains about 50% at 5 years of PAH despite the application of targeted drugs (Thenappan et al., 2018). Mechanistically, renal dysfunction, vascular dysfunction, and central nervous system disorder are critically involved in the pathogenesis of arterial hypertension (Ren et al., 2020; Zhang and Sun, 2020; Harrison et al., 2021). Mutations in the type II bone morphogenetic protein receptor (BMPR2), chronic inflammation, fibrosis, immune activation, and mitochondrial metabolic dysfunction are drivers for the pathogenesis of PAH (Thenappan et al., 2018). Overall, the pathophysiology of arterial hypertension and PAH is heterogeneous and multifactorial, thus, it is of great significance to comprehensively understand the pathogenesis of arterial hypertension and PAH. Despite current therapies, such as renin-angiotensin system blockers, calcium antagonists, steroidal mineralocorticoid receptor antagonists, and thiazide-type diuretic, almost half of hypertensive patients are not sufficiently controlled (Bakris et al., 2020). Optimizing the prevention, treatment, and recognition of hypertension requires a better understanding of the pathogenesis network of hypertension.

It is currently accepted that vascular remodeling is a characteristic during the development and progression of hypertension since hypertension is a driving force for endothelial cell activation, vascular smooth muscle cells (VSMCs) and adventitial fibroblasts dysfunction (Zhu et al., 2018; Zhang and Sun, 2021). Conversely, vascular remodeling/stiffening is an important pathological basis for maintaining high blood pressure, thus forming a vicious circle (Kostov, 2021). Specifically, the imbalanced vasoactive factors produced by the endothelium induce vasoconstriction, proinflammatory state, oxidative stress and deficiency of nitric oxide (NO), a critical event implicated in the pathophysiology of hypertension (Zhang and Sun, 2020). The anomalous apoptosis, phenotype conversion, proliferation, and migration of VSMCs are related to the progression of hypertension-induced vascular remodeling (Zhang and Sun, 2020). As important components of blood vessels, adventitial fibroblasts are essential for vascular homeostasis and their malfunction plays an important role in aortic maladaptation and hypertension-related vascular fibrosis (Ling et al., 2018; Tong et al., 2018). Animal and cellular experiments support the therapeutic potential of vascular remodeling reversal in hypertension and its related organ damage (Ouarné et al., 2021). Thus, a better understanding of the etiology of vascular remodeling might open new therapeutic approaches for hypertension.

Recently, nonpharmacological interventions are highly proposed for adults with elevated blood pressure or hypertension, and alternative therapies are therefore becoming an adjuvant treatment of hypertension (Arnett et al., 2019; Canale and Noce, 2021). In particular, phytochemicals are emerging as alternative therapies with therapeutic effects on a wide range of pathologies, including hypertension. Of those phytochemicals, curcumin is a highly pleiotropic molecule with anti-inflammatory, antioxidant, chemosensitizing, neuroprotective, renoprotective, hepatoprotective, lipid-modifying, glucose-lowering, anti-atherogenic effects (Hadi et al., 2019). Coincidentally, curcumin is found to exert anti-hypertensive actions through a broad spectrum of targets or signaling pathways (Hadi et al., 2019; Cox and Misiou, 2022). For example, supplementation of curcumin is able to improve PAH through reversing pulmonary vessel remodeling and fibrosis (Lin et al., 2006). Inhibition of nitric oxide synthesis (NOS) with N(ω)-nitro-L-arginine methyl ester (L-NAME) elevates arterial blood pressure and peripheral vascular resistance in male Sprague-Dawley rats, an effect that is largely mitigated by curcumin or hexahydrocurcumin treatment (Nakmareong et al., 2011; Nakmareong et al., 2012; Panthiya et al., 2022). Supplementation of curcumin reduces blood pressure and attenuates vascular oxidative stress and structural modifications in 2kidney-1clip (2K1C)-induced hypertensive rats (Boonla et al., 2014). Curcumin gavage attenuates 5/6 nephrectomy-induced systemic and glomerular hypertension, hyperfiltration, glomerular sclerosis, and interstitial fibrosis, which is associated with increased nuclear translocation of nuclear factor erythroid 2-related factor 2 (Nrf2) (Tapia et al., 2012). In a mouse model of cadmium-induced hypertension, curcumin is found to protect against vascular dysfunction through upregulation of endothelial nitric oxide synthase (eNOS) protein, restoration of glutathione redox ratio, and attenuation of oxidative stress (Kukongviriyapan et al., 2014). Similar to this finding, supplementation with curcumin significantly reduces blood pressure, alleviates oxidative stress, and increases plasma nitrate/nitrite and glutathione in the blood from rats with chronic exposure to lead and cadmium (Tubsakul et al., 2021). In addition, treatment with curcumin prevents cardiac dysfunction and heart failure in salt-sensitive Dahl rats, independently of hypertension (Morimoto et al., 2008; Sunagawa et al., 2021). We have revealed that curcumin exhibits anti-hypertensive effects in spontaneously hypertensive rats (SHR) by suppressing vascular inflammation and remodeling (Sun H.-J. et al., 2017; Han et al., 2019). Very recently, a study by Kang’s group has shown that curcumin reshapes the composition of the gut microbiota to grant antihypertensive effects (Li et al., 2021). These published papers provide ample evidence that curcumin is a promising protective agent against vascular dysfunction induced by PAH and arterial hypertension, such as primary hypertension, renovascular hypertension, salt sensitive hypertension, L-NAME-induced hypertension, angiotensin II (Ang II)-induced hypertension, 5/6 nephrectomy-induced systemic hypertension, and cadmium-induced hypertension. In the present review, we will recapitulate the cellular and molecular mechanisms that are responsible for curcumin-induced protection against hypertension and vascular remodeling. We further highlight the controversies and inconsistencies of curcumin in clinical settings. Eventually, we try to propose how addressing the bioavailability and pharmacokinetics of curcumin could help to prevent or treat hypertension-related vascular disorders.

## Aberrant Vascular Cell Behaviors in Arterial Hypertension and Pulmonary Arterial Hypertension

**FIGURE 1:**
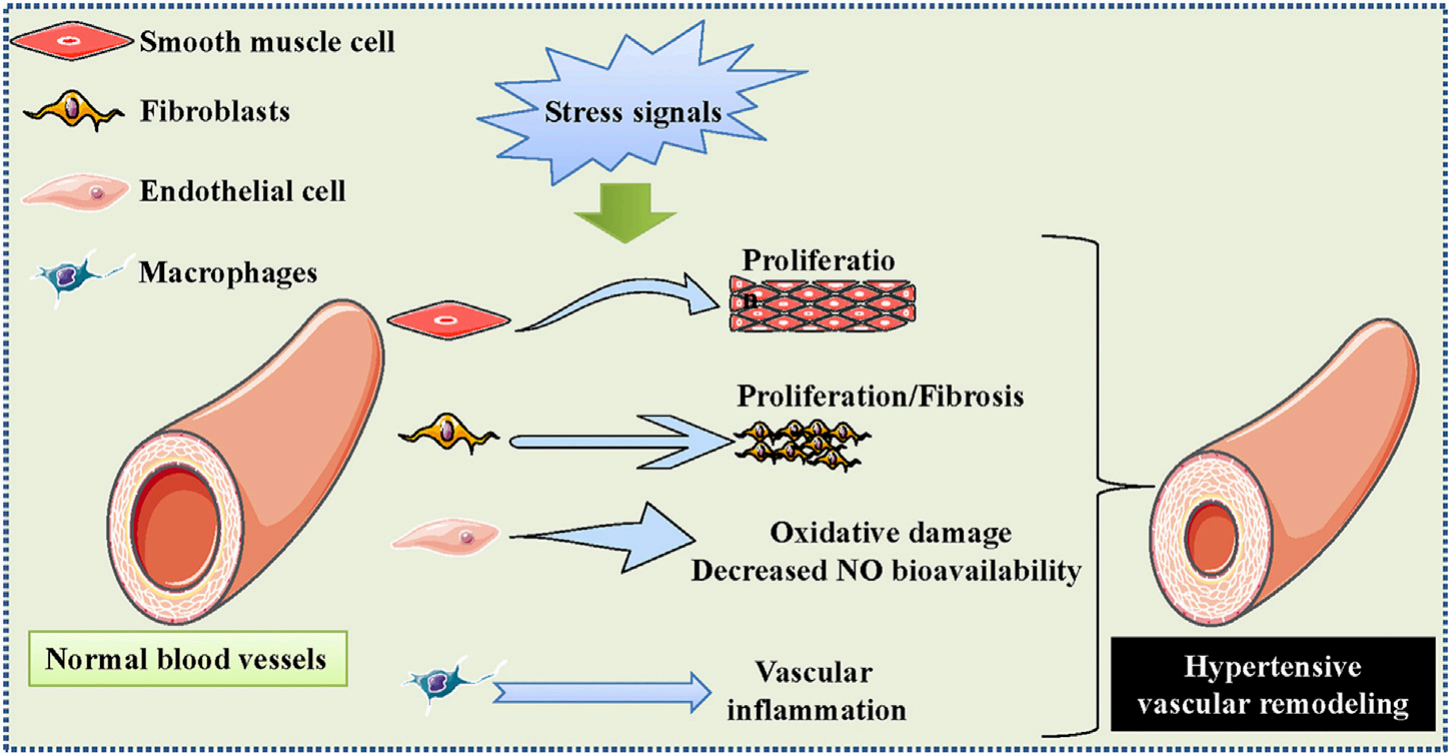
Schematic presentation regarding the role of vascular dysfunction in hypertension. The blood vessels are constituted of VSMCs, endothelial cells, fibroblasts, extracellular matrix, and inflammatory cells, which are mandatory for vascular homeostasis. The excessive proliferation and migration of VSMCs, increased oxidative stress and impaired NO bioavailability in the endothelium, adventitial fibroblast activation, as well as macrophages-mediated vascular inflammation are important contributors to the development of hypertensive vascular remodeling. VSMCs, vascular smooth muscle cells; NO, nitric oxide.

The blood vessels are constituted of VSMCs, endothelial cells, adventitial fibroblasts, and extracellular matrix, and these components are mandatory for vascular homeostasis (Kim et al., 2019). It is well known that hypertension is intricately linked to large and small vascular remodeling impacting cardiovascular outcomes and prognosis (Briet and Schiffrin, 2013). Resistance small arteries are crucial players in the regulation of systemic blood pressure, and a functional reduction in small arteries is an early event in the initiation and progression of hypertension (De Ciuceis et al., 2007). The hypertrophic remodeling in resistance small arteries is detected in hypertension, which is characterized by an increase in the wall cross-sectional area, media-to-lumen ratio, and a decrease in internal diameter (Boari et al., 2010). These structural abnormalities are closely linked to enhanced vasoconstrictor response and progressive extracellular matrix deposition (Schiffrin et al., 1993; Rizzoni et al., 2000). The elastic properties of large arteries play a necessary role in the ventricular-aortic coupling, and such arteries are important determinants of systolic blood pressure (Briet and Schiffrin, 2013). The structural impairment of large arteries, such as collagen deposition and elastin fragmentation, leads to increased thickness and stiffness of the large arterial wall (Briet and Schiffrin, 2013). Elevated aortic stiffness is closely related to increased risk of cardiovascular events in hypertensive patients (Blacher et al., 1999; Aryal and Siddiqui, 2021). In the context of hypertension, vascular cells undergo cell proliferation, migration, and death, contributing to increased vascular thickness and stiffness, along with decreased compliance of blood vessels (Figure 1) (Brown et al., 2018). Specifically, VSMCs might exhibit phenotypic conversion from a differentiated to a dedifferentiated condition, which is a pathological hallmark of hypertension-triggered vascular remodeling (Laurent and Boutouyrie, 2015; Lu et al., 2018b). Endothelial cell inflammation and oxidative stress, increased endothelial cell permeability, impaired NO bioavailability, and destructed endothelial-dependent vasodilatation, are crucial initiating factors for blood pressure elevation (Sun et al., 2016; Sun HJ. et al., 2019). Activation of adventitial fibroblasts plays a critical role in the overproduction and deposition of collagens around the blood vessels, leading to vascular fibrosis and remodeling in hypertension (Ling et al., 2018; Tong et al., 2018; Gumprecht et al., 2019; Ren et al., 2020; Wu et al., 2020; Tong et al., 2021). It is noteworthy to mention that communications between blood vessel cells are responsible for the development of hypertensive-related vascular remodeling (Sun HJ. et al., 2017; Zhang and Sun, 2020; Zhang and Sun, 2021). There is no doubt that impaired vascular cell function is one of the pathological characteristics for the development of arterial hypertension.

PAH-related vascular remodeling is associated with thickening of the tunica intima and tunica media (Humbert et al., 2008). The proliferation of pulmonary arterial endothelial cells and exuberant angiogenesis results in the formation of glomeruloid-like plexiform lesions, a common pathological feature of the pulmonary vessels of PAH patients (Tuder, 2017). Apart from this, changes in the production of various endothelial vasoactive molecules, including NO, prostacyclin, endothelin-1 (ET-1), serotonin, chemokines and thromboxane, play an important role in regulating the structural/functional alterations in the pulmonary vasculature (Tuder et al., 2013). It is convincing that excessive growth of pulmonary VSMCs could facilitate the development and progression of pulmonary vascular hypertrophy and structural remodeling in PAH (Jeffery and Morrell, 2002). Moreover, the structural changes in the intima, media and adventitia of pulmonary vessels contribute to a decrease in lumen diameter and reduced capacity for vasodilatation (Perros et al., 2005). In an autopsy series of 19 patients with PAH, an approximately 2- to 4-fold increase in adventitia thickness is observed in PAH lungs (Chazova et al., 1995). There is substantial evidence that the pulmonary arterial adventitia might function as an inflammatory cell signaling hub to boost pulmonary vascular remodeling (Pugliese et al., 2015; Stenmark et al., 2015). Apart from vascular cells, inflammatory cells and platelets may also play an essential role in the etiologies of arterial hypertension and PAH (Stumpf et al., 2016; Tuder, 2017), this deserves further studies. Collectively, in-depth elucidation of the intimate relationship between hypertension and vascular remodeling might permit novel therapeutic interventions of arterial hypertension and PAH.

### Curcumin Provides Vascular Protection in Arterial Hypertension and Pulmonary Arterial Hypertension

As a yellow-colored hydrophobic polyphenol, curcumin is the main ingredient of spice turmeric with anti-inflammation, anti-carcinogenesis, anti-obesity, anti-angiogenesis, and anti-oxidant activities (Shishodia et al., 2005; Alappat and Awad, 2010; Meydani and Hasan, 2010). Accumulative evidence has demonstrated that curcumin is capable of preventing the development and progression of hypertension. For example, a study by Nakmareong et al. (2011) has revealed that administration of curcumin suppresses the elevation of blood pressure, decreases vascular resistance, and restores vascular responsiveness in L-NAME-infused rats. A later study by the same group has found that application of tetrahydrocurcumin, a major metabolite of curcumin, improves hypertension, along with reduced aortic wall thickness and stiffness in rats after L-NAME administration (Nakmareong et al., 2012). Oral gavage of curcumin prevents the elevation of blood pressure, diminishes the increased wall thickness and cross-sectional area of the aorta in experimental L-NAME-induced hypertensive rats (Hlavačková et al., 2011). Kukongviriyapan et al. (2014) have disclosed that both curcumin and tetrahydrocurcumin are effective in ameliorating cadmium-evoked hypertension and vascular dysfunction in mice (Kukongviriyapan et al., 2014; Sangartit et al., 2014). In agreement with these results, oral administration of hexahydrocurcumin, another major metabolite of curcumin, possesses antihypertensive actions by inhibiting vascular inflammation and oxidative stress, and activating the eNOS/NO pathway in aortic tissues, thus ameliorating vascular remodeling in hypertensive rats induced by L-NAME (Panthiya et al., 2022). As a result, it is clear that curcumin and its derivatives might provide a valuable way for the treatment of arterial hypertension or cadmium-induced hypertension.

It has been well established that curcumin treatment inhibits Ang II-induced hypertension and vasoconstriction, an effect that is mediated by downregulation of Ang II type 1 receptor (AT1R) expressions in the arteries (Yao et al., 2016). The elevated blood pressure and myocardial fibrosis are detected in male Sprague-Dawley rats subjected to Ang II infusion, whereas these abnormal changes are corrected by gastric gavage of curcumin (Pang et al., 2015). The myocardial protein level of AT1R is reduced, and the Ang II type 2 receptor (AT2R) expression is upregulated in curcumin-treated rats when compared to Ang II-infused rats (Pang et al., 2015). Furthermore, curcumin upregulates the protein level of angiotensin-converting enzyme 2 (ACE2) in the intermyocardium relative to the Ang II-treated rats (Pang et al., 2015). After 6 weeks of treatment, curcumin reduces blood pressure and vascular resistance in 2K1C-induced hypertensive rats, which is accompanied by attenuation of vascular structural modifications and oxidative stress (Boonla et al., 2014). A systematic review and meta-analysis has shown that long-term consumption of curcumin might improve systolic blood pressure, without affecting diastolic blood pressure (Hadi et al., 2019).

The clinical evolution of PAH results in a progressive debilitation, greatly reducing the quality of life in patient. Although the available drugs, such as prostanoids, phosphodiesterase inhibitors and antagonists of ET-1 for PAH therapy, the current therapeutic approaches are few and expensive. On the basis of the anti-inflammatory effects of curcumin, (Bronte et al., 2013) hypothesized a therapeutic role of curcumin or its derivatives for PAH (Bronte et al., 2013). Afterwards, curcumin and its analogues are reported to reverse the development of PAH and pulmonary vascular remodeling by preserving mitochondrial function in VSMCs (Chen et al., 2021), and retarding pulmonary fibrosis (Li et al., 2014). A series of curcumin analogues dilate rat pulmonary arteries via inhibiting phosphodiesterase-5 (PDE5) activities, suggesting that selective inhibition of PDE5 by curcumin may be a promising strategy for the prevention and treatment of PAH (Kruangtip et al., 2015). The Matrigel migration assay demonstrated that curcumin effectively prevented tumor necrosis factor-α (TNF-α)-induced migration of human aortic smooth muscle cells by suppressing matrix metalloproteinase 9 (MMP-9) expression through downregulation of the nuclear translocation of NF-κB p50 and p65 (Yu and Lin, 2010). These above findings hint that curcumin may be a promising candidate for the prevention and treatment of hypertension. Despite the exciting results, the hypertension-lowering effects of curcumin should be treated with caution and more preclinical and clinical studies are warranted to ascertain these results.

The immune cell infiltrate is observed in blood vessels from arterial hypertension and PAH, including macrophages (Rabinovitch et al., 2014; Foley et al., 2021). Preclinical studies have provided that macrophage dysfunction underlies the development of vascular inflammation and remodeling in hypertension (Huo et al., 2021). Macrophages are phenotypically heterogeneous in which M1- and M2-type macrophages bear different features and functions (Stöger et al., 2012). A significant decrease of M2 macrophage markers and a marked increase of M1 macrophage markers play a pivotal role in hypertensive remodeling through numerous molecular mechanisms (Luo and Qiu, 2022), indicating that the pro-inflammatory status of macrophages drives the pathogenesis of hypertensive vascular remodeling. It is not surprising that curcumin could regulate macrophage functions in chronic inflammatory diseases (Mohammadi et al., 2019). It is reported that curcumin dose-dependently inhibits M1 macrophage polarization through downregulating toll-like receptor 4 (TLR4)-mediated activation of ERK, JNK, p38, and nuclear factor (NF)-κB (Zhou et al., 2015). In similarity with this finding, a curcumin derivative 2,6-bis(2,5-dimethoxybenzylidene)-cyclohexanone (BDMC33) is documented to suppress the secretion of pro-inflammatory mediators in stimulated macrophages via inhibition of NF-κB and MAPK signaling pathways, as well as suppression of prostaglandin E2 (PGE2) and cyclooxygenase (COX) expressions (Lee et al., 2011; Lee et al., 2012). Besides, administration of nano-emulsion curcumin blocks the phosphorylation of p65 NFκB and IκBα in macrophages induced by lipopolysaccharide (LPS), and reduces macrophage recruitment in a mouse model of peritonitis (Young et al., 2014). These findings suggest that curcumin could be used as a therapeutic agent in the management of hypertensive vascular remodeling by regulating macrophage polarization and activation, and curcumin may hold clinical promise for the prevention and treatment of hypertension due to its anti-inflammatory and immunomodulatory actions, especially considering that hypertension is also a chronic inflammatory disease (Sun H.-J. et al., 2017).

### Curcumin Provides Vascular Protection in Hypertension Caused by Cadmium

Cadmium, a nonessential heavy metal, is documented to cause oxidative stress in various organs and tissues associated with hypertension (Pinheiro Júnior et al., 2020). A study by Greenwald’s group showed that exposure of cadmium chloride results in hypertension, hypertrophic aortic wall, blunted vasodilation, increased aortic stiffness, collagen deposition, and accumulation of MMP-2 and MMP-9 levels in the aortic medial wall of male ICR mice, effects are largely ameliorated by tetrahydrocurcumin, a major metabolite of curcumin (Sangartit et al., 2014). The signaling mechanisms of tetrahydrocurcumin are shown to be associated with eNOS activation, enhanced antioxidant glutathione, decreased nitrate/nitrite level and oxidative stress in vascular tissues (Sangartit et al., 2014). Kukongviriyapan et al. found that intragastric administration of curcumin protected vascular dysfunction by increasing vascular responsiveness to acetylcholine, as well as normalizing the blood pressure elevation in cadmium chloride-challenged mice, a phenomenon that was eNOS-dependent (Kukongviriyapan et al., 2014). Exposure to lead and cadmium results in increases in blood pressure and peripheral vascular resistance, with a concomitant decrease in the blood pressure response to intravenous infusion to acetylcholine in male Sprague-Dawley rats (Tubsakul et al., 2021). By contrast, supplementation with curcumin effectively reduces blood pressure, alleviates oxidative stress, ameliorates vascular responsiveness through upregulation of eNOS and downregulation of the nicotinamide adenine dinucleotide phosphate (NADPH) oxidase expressions in the blood vessels of rats exposed to lead and cadmium (Tubsakul et al., 2021), an observation that supports the potential of curcumin as a candidate in the treatment of hypertension induced by the heavy metals. To this end, these findings consistently imply that curcumin might serve as a promising agent against hypertension and vascular remodeling induced by cadmium chloride due to its antioxidant, anti-nitrative, and chelating properties, which had also been meticulously introduced in a review (Kukongviriyapan et al., 2016). More experimental studies and clinical trials are required to replicate the benefit of curcumin and its active metabolites in hypertension and vascular remodeling upon cadmium chloride exposure.

### Curcumin Nanomedicine in Hypertension

Although curcumin has been widely used for the treatment of various diseases, its unfavorable pharmacokinetics and pharmacodynamics profiles, including poor water solubility, poor bioavailability, and short biological half-life time, might hamper its biomedical and/or clinical applications (Pathak and Khandelwal, 2008). Indeed, approximately 60%–70% of oral administered curcumin is not absorbed due to their rapid hydrolyzation at physiological pH (Tønnesen et al., 2002; Anand et al., 2007). To resolve this obstacle, the microparticle-based systems are applied to enhance the bioavailability of curcumin (Shahani et al., 2010; Shome et al., 2016). Thus, the field of curcumin nanomedicine is flourishingly emerging (Pechanova and Dayar, 2020; Hesari and Mohammadi, 2021). To date, a number of nanomedicine-based drug delivery systems are used for curcumin delivery, such as mesoporous silica nanoparticles, exosomes, nanoemulsions, cyclodextrin inclusion complexes, nanogels, carbon nanotubes, liposomes, solid lipid nanoparticles, dendrosomes, dendrimers, polymeric nanoparticles, silver and gold nanoparticles, micelles, niosomes, nanocrystals, and nanosuspensions (Salehi and Del Prado-Audelo, 2020). These promising approaches provide a solid platform to figure out the problems of curcumin delivery (Ahangari et al., 2019). Nanotechnology-mediated curcumin delivery formulation is used to treat a wide range of diseases, such as diabetes (Maradana et al., 2013), wound healing (Hussain et al., 2017), inflammatory diseases (Yallapu et al., 2015), neurodegenerative disorders (Rakotoarisoa and Angelova, 2018), and cancers (Yallapu et al., 2012). Nanocurcumin is also used to prevent and treat hypertension and its complications through improving its aqueous-phase solubility and bioavailability in the target tissues (Shome et al., 2016).

Compared to monocrotaline (MCT)-induced PAH rats, the right ventricular wall thickness and right ventricle weight/body weight ratio were largely reduced in PAH rats treated with curcumin nanoparticles (Rice et al., 2016). Besides, curcumin nanoparticles treatment attenuated MCT-induced expressions of TNF-α, interleukin 1β (IL-1β), nitrotyrosine, fibronectin, and myosin heavy chain-β in the right ventricle tissues (Rice et al., 2016). The delivery of curcumin to the vascular wall using hyaluronic acid-based nanocapsules caused a gradual inhibition of systolic blood pressure, diastolic blood pressure, and mean arterial pressure in hypertensive TGR(m-Ren2)27 rats (Czyzynska-Cichon and Janik-Hazuka, 2021). Surprisingly, administration of a curcumin solution (4.5 mg/kg) had no hypotensive effect in these animals (Czyzynska-Cichon and Janik-Hazuka, 2021), suggesting that hyaluronic acid-based nanocapsules might serve as a suitable approach to deliver hydrophobic and poorly bioavailable curcumin to the vascular wall (Czyzynska-Cichon and Janik-Hazuka, 2021). Curcumin nanoparticles encapsulated in poly(lactic-co-glycolic acid) (5 mg/kg/day) are found to normalize blood pressure and cardiovascular remodeling in male Wistar rats with diet-induced metabolic syndrome, an observation that is similar to the effects of unformulated curcumin (100 mg/kg/day) (Du Preez et al., 2019), implying that nanoparticle formulations might allow low-dose of curcumin to confer its benefits to health. Curcumin acetate nanocrystals significantly increase the pulmonary absorption time by 7.2-fold when compared to the sole curcumin, possibly because of the high lipophilicity of the former (Hu et al., 2017). This system achieves a better pharmacological efficacy in a MCT-induced rat model of PAH (Hu et al., 2017). A significant increase in physical properties, bioavailability, and stability is observed when curcumin is encapsulated in a nanoemulsion, and curcumin nanoemulsion shows significant anti-hypertensive and cholesterol-lowering activities, indicating the improved solubility of curcumin in the nanoemulsion system (Rachmawati et al., 2016). As aforementioned, curcumin nanoparticles provide feasible strategies for the sustained delivery of curcumin in a new discovery phase, thus improving its bioavailability and efficiency.

Despite having huge potential benefits of curcumin nanomedicine, the anti-hypertensive effects of curcumin nanomedicine are challenged by a study that encapsulation of curcumin in biodegradable poly(lactide-co-glycolic) acid nanoparticles failed to grant any protection against hypoxia-induced PAH in experimental animals (Devadasu et al., 2012). This study indicated that hypoxia conditions might affect the localization of particles in the lungs, which may be due to blood flow changes, increased barrier properties of the pulmonary vascular system, and decreased endocytosis (Devadasu et al., 2012). The tissue levels of curcumin under hypoxia circumstances are much lower than that of normoxic conditions because of the difference in particle dynamics, leading to failure of PAH treatment (Devadasu et al., 2012). Despite these contradictory results, more preclinical studies with rigorous experimental designs are warranted to verify the efficacy of nanomaterials-mediated curcumin transfer in hypertension. To sum up, nanomedicine bridges the gap between pharmaceutical limitations and the therapeutic potentials of curcumin via strengthening curcumin’s pharmacokinetics, efficacy, and cellular uptake.

## Molecular Mechanism of Curcumin Improving Vascular Remodeling in Hypertension

Next, the cellular and molecular mechanisms of actions of curcumin and its derivatives in hypertension and vascular remodeling will be critically described. In short, curcumin might benefit hypertension and vascular remodeling *via* multiple mechanisms, such as suppression of vascular contraction, inhibition of VSMC proliferation and migration, amelioration of endothelial cell dysfunction, and blockade of the renin angiotensin system (RAS), etc.

## Suppression of Vascular Contraction

Elevated vascular resistance is one of the main pathological events in the development and maintenance of hypertension, paralleling by abnormal vasoconstriction, impaired vasodilation, and vascular remodeling (Jouen-Tachoire and Tucker, 2021; Mccoy and Lisenby, 2021; Prado et al., 2021). VSMC tone is mainly regulated by changes in both intracellular Ca^2+^ concentration and myofilament Ca^2+^ sensitivity, which is enhanced by L-type Ca^2+^ channels in the membrane of VSMCs and inositol 1,4,5-trisphosphate (IP3)-mediated Ca^2+^ release from sarcoplasmic reticulum via G protein-coupled receptor-induced phospholipase C activation (Karaki and Weiss, 1988; Karaki et al., 1997). Under physiological states, the L-type Ca^2+^ channels are slowly inactivated in the process of continuous depolarization, and the influx of Ca^2+^ is sufficient to mediate pressure-induced vasoconstriction of resistance vessels, thus contributing to the dynamic auto-regulation of systemic arteries (Nelson et al., 1990). However, the occurrence of abnormal arterial tension is closely linked with increased expressions of L-type Ca^2+^ channel α1C subunits, resulting in increasing blood pressure and flow (Wang et al., 1999). Therefore, L-type Ca^2+^ channel antagonists could be expected to act as adjuvants for anti-hypertensive agents as L-type Ca^2+^ channel overexpression is not limited to hypertension (Cox and Rusch, 2002). ET-1, a vasoactive peptide of 21-amino acids, is known as one of the most potential vasoconstrictors, which plays a critical role in the development of hypertension by acting on endothelin receptor type A (ET_A_R), type B1 (ET_B1_R), and type B2 (ET_B2_R) coupled with G proteins (Sakurai et al., 1990; Kedzierski et al., 2003). ET_A_R and ET_B2_R mainly mediate vasoconstriction and cell proliferation, whereas ET_B1_R have vasodilatation, anti-inflammatory, and ET-1 peptides-clearing functions (Webb and Meek, 1997; Lüscher and Barton, 2000). Increased ET-1 expressions and altered expressions of ET-1 receptors are well characterized in hypertension (Wang B. et al., 2021). Accordingly, the antagonists of ET-1 or inhibitors of ET_A_/ET_B2_ receptor are clinically important to prevent or treat cardiovascular illnesses including essential hypertension and PAH (Barton and Yanagisawa, 2019; Dhaun and Webb, 2019; Xiao et al., 2021). To this end, it is important to develop novel vasodilation drug candidates that affect both L-type Ca^2+^ channel- and ET-1-induced vascular constriction, thus advancing the development of antihypertensive drugs. Coincidentally, Park et al. (2015) have identified alkylsulfonyl and substituted benzenesulfonyl curcumin mimics as a dual antagonist of L-type Ca^2+^ channel and endothelin A/B2 receptor in VSMCs, representing them as potential drug candidates to treat hypertension and vascular remodeling. Also, circulating ET-1 plays a central role in regulating renal electrolyte and water handling (Tamás et al., 1994; Bomzon et al., 1997; Ota et al., 1998; Lynch et al., 2015), since intravenous infusion of ET-1 profoundly increases renal vascular resistance and decreases the excretion of sodium and water (Sørensen et al., 1995). Selective blockade of ET_A_R and ET_B_R differentially affects renal tubular water and salt handling in Wistar-Kyoto and Long-Evans rats (Girchev et al., 2006). On the contrary, centrally administered ET-1 had no effect on renal handling of water and electrolytes (Yamamoto et al., 1991). Given the role of ET-1 in renal electrolytes and water handling, and the opposed effects of curcumin on ET-1-mediated effects, it will be of importance to investigate whether curcumin lowers hypertension by affecting renal handling of water and electrolytes and renovascular contraction.

The exaggerated vasoconstrictor generation by cyclooxygenase-2 (COX-2) is a driving force for the development of hypertension-related vascular contraction (Virdis and Taddei, 2016). Thus, it is interesting to test whether curcumin conserves vascular function in hypertension by inhibiting COX-2 production. Accordingly, (Li and Tian, 2016) examined this hypothesis and found that demethoxycurcumin, a major component of *Curcuma longa* L, elevated endothelium-dependent contractions in renal arteries of SHR by normalization of COX-2 expression, indicating the benefits of demethoxycurcumin in endothelium-dependent contractions during the development of hypertension (Li and Tian, 2016).

Malfunction in vascular reactivity is an important component of diabetes-related hypertension (Farhangkhoee et al., 2003). Curcumin is reported to attenuate phenylephrine-induced increase in contraction during the early stage of streptozotocin-induced diabetic rats, suggesting a regulatory role of curcumin in cardiometabolic diseases-associated vascular dysfunction (Majithiya and Balaraman, 2005). Zakaria’s group has uncovered that curcumin treatment reduces elevated systolic blood pressure and prevents aorta-exaggerated response to phenylephrine and potassium chloride (KCl) in diabetes-evoked hypertensive rats (Hassan et al., 2013; El-Bassossy et al., 2014). Further studies have suggested that curcumin functions as a heme oxygenase-1 (HO-1) inducer to protect against exaggerated vascular contractility by reducing TNF-α and aortic reactive oxygen species (ROS) levels (Hassan et al., 2013). In consistence with this finding, exogenous curcumin effectively abrogates high fructose-elicited contractile response of aortic rings to both phenylephrine and KCl through reduction of intracellular ROS and calcium (Mahmoud and El Bassossy, 2014). Overall, these published papers imply that curcumin acts as a promising candidate to suppress hypertension-induced vasoconstriction. In spite of this, more studies are necessary to affirm these observations, thus pointing towards that the vascular benefits of curcumin may be dependent on circumventing vasoconstriction in hypertension. Here, it should be mentioned that renal artery narrowing is a major driver for hypertensive development by producing renal vascular resistance from the glomerulus (Anderson et al., 2000). Increased renal arterial contraction and renovascular resistance are also closely associated with the pathologies of hypertension (Restini et al., 2018). Although the renoprotective roles of curcumin in hypertension (Boonla et al., 2014; Yaribeygi et al., 2021), it remains to be explored whether amelioration of renal function or inhibition of renovascular contraction contributed to the blood pressure lowering actions of curcumin.

## Inhibition of Vascular Smooth Muscle Cell Proliferation and Migration

**FIGURE 2:**
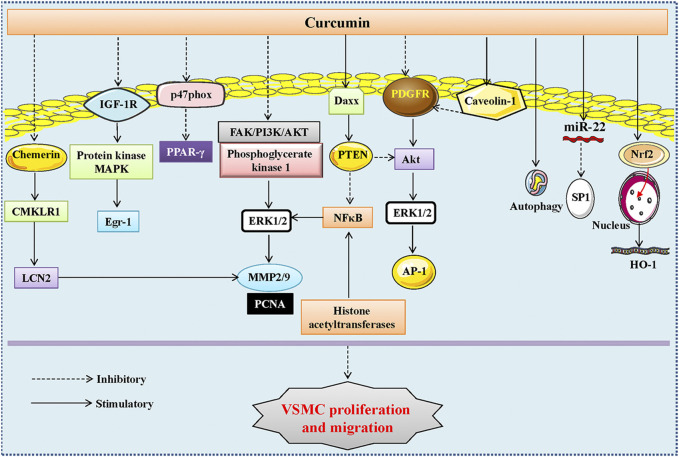
The signaling pathways involved in the inhibitory action of curcumin in VSMC proliferation and migration. The inhibitory effects of curcumin and its analogue or derivatives in VSMC proliferation and migration were associated with inhibition of the FAK/PI3K/AKT and phosphoglycerate kinase 1/ERK1/2 signaling pathways, blockade of the protein kinase and mitogen-activated protein kinase (MAPK) and the p38 MAPK and Wnt/β-catenin signaling pathways, suppression of histone acetyltransferases and PDGF receptor-β phosphorylation, Akt and ERK1/2, inactivation of the IGF-1R/PKB/ERK1/2/Egr-1 axis, regulation of the PTEN/Akt pathway and the miR-22/SP1 axis, repression of the chemerin/CMKLR1/LCN2 signaling pathway, activation of caveolin-1, Nrf2/HO-1, PPAR-γ, autophagy, and Daxx. VSMCs, vascular smooth muscle cells; FAK, focal adhesion kinase; PI3K, phosphatidylinositol 3-kinase; AKT, protein kinase B; ERK1/2, extracellular signal regulated kinase 1/2; IGF-1R, insulin-like growth factor type 1 receptor; Egr-1, early growth response; PPAR-γ, peroxisome proliferator-activated receptor-γ; CMKLR1, chemokine-like receptor 1; LCN2, lipocalin-2; Nrf2, nuclear transcription factor E2-related factor-2; HO-1, heme oxygenase-1; PTEN, phosphatase and tensin homolog; PDGF, platelet-derived growth factor; SP1, specificity protein 1.

Phenotypic switching, a prerequisite step for abnormal proliferation and migration of VSMCs, is characterized by a transition from a contractile phenotype (differentiated phenotype) to a synthetic phenotype (dedifferentiated phenotype) under various stimuli, including hypertension (Shi and Chen, 2014). Excessive proliferation and migration of VSMCs are important characteristics of hypertension, and reversing this process might be an important strategy to fight against hypertension and its associated vascular remodeling (Harrison et al., 2021). In light of the anti-hypertensive effects of curcumin, it is not unexpected to observe that curcumin might benefit hypertension by regulating the biological behaviors of VSMCs (Figure 2).

A previous report had shown that curcumin does-dependently inhibited the proliferation of rabbit VSMCs stimulated by fetal calf serum (Huang et al., 1992), indicating that curcumin is a promising remedy for the prevention of the pathologies of vascular remodeling-related diseases, such as atherosclerosis, restenosis, and hypertension. A later study further demonstrated that curcumin prevented cell proliferation, arrested the cell cycle progression, and facilitated cell apoptosis in VSMCs (Chen and Huang, 1998). Mechanistic studies have shown that the antiproliferative effects of curcumin may be mediated by inhibition of protein tyrosine kinase activity and c-myc mRNA expression, while the apoptotic effects of curcumin might partly be modulated by suppression of protein tyrosine kinase activity, protein kinase C activity, c-myc mRNA expression and B-cell lymphoma-2 (Bcl-2) mRNA expression (Chen and Huang, 1998). Demethoxycurcumin, a major active curcuminoid from Curcuma longa, exhibits similar actions on fetal calf serum-stimulated VSMC proliferation and migration by downregulating the expression of MMP-2 and MMP-9 via dampening the focal adhesion kinase (FAK)/phosphatidylinositol 3-kinase (PI3K)/AKT (protein kinase B) and phosphoglycerate kinase 1/extracellular signal regulated kinase 1/2 signaling pathways (Sheu et al., 2013). Moreover, blockade of the protein kinase and mitogen-activated protein kinase (MAPK) pathways was also required for curcumin to restrain VSMC proliferation, inflammation, and oxidative stress (Nguyen et al., 2004). Collectively, these findings indicate that curcumin and its analogues can be developed to inhibit VSMC proliferation and migration through various signaling pathways.

A number of growth factors, such as platelet-derived growth factor (PDGF) and fibroblast growth factor, are responsible for the proliferation and migration of VSMCs, an event in the pathological changes of hypertension and vascular injury (Heldin and Westermark, 1999; Lu et al., 2018a). Consequently, blockade of PDGF signaling may represent an important avenue to restrain VSMC dedifferentiation, migration, proliferation, and extracellular matrix synthesis during the process of hypertension and restenosis after vascular injury. In this regard, Yang et al. examined the effects of curcumin on PDGF-stimulated VSMC proliferation and migration, and found that curcumin evoked a concentration-dependent inhibition of VSMC migration, proliferation, and collagen synthesis in PDGF-incubated VSMCs (Yang et al., 2006). Animal studies showed that carotid artery neointima formation was strikingly attenuated by perivascular administration of curcumin (Yang et al., 2006). Similar to this finding, dehydrozingerone, a structural analog of curcumin, induces a dose-dependent inhibition of PDGF-stimulated VSMC migration, proliferation, collagen synthesis *via* inhibiting the phosphorylation of PDGF-receptor (PDGFR) and AKT (Liu et al., 2008). Additionally, the anti-proliferative effects of curcumin on PDGF-induced VSMC proliferation are caveolin-1-dependent as disruption of caveolae with methyl-β-cyclodextrin eliminates curcumin-mediated effects, suggesting that upregulation of caveolin-1 is implicated in curcumin-induced benefits in the vasculature (Zeng et al., 2013). Bisdemethoxycurcumin, a naturally occurring structural analog of curcumin, induces a concentration-dependent inhibition of PDGF-stimulated VSMC migration and proliferation *via* impeding the phosphorylation of PDGF receptor-β, AKT and ERK (Hua et al., 2013). Epidermal growth factor (EGF) acts on epidermal growth factor receptor (EGFR) to trigger numerous signaling cascades leading to the proliferation and migration of VSMCs. A host of transcription factors contribute to EGFR overexpression, including activator protein (AP-1). An interesting study showed that curcumin had the ability to halt VSMC proliferation by functioning as an AP-1 inhibitor (Hsieh et al., 2008a; Hsieh et al., 2008b). A better understanding of the underlying mechanisms involved in curcumin-regulated AP1 expression in VSMCs may offer potential targets in the treatment of hypertensive vascular remodeling. These findings provide evidence that the anti-proliferative effect of curcumin is largely linked to its ability to mitigate the PDGF and EGFR signaling pathways.

Phosphatase and tensin homolog (PTEN) is a well-recognized tumor suppressor gene that plays a fundamental role in the proliferation and migration of VSMCs, thus representing a potential target to treat vascular remodeling. HO-3867, a novel synthetic curcuminoid, is shown to inhibit the proliferation of serum-stimulated VSMC proliferation through activation of PTEN, followed by downregulation of MMP-2, MMP-9, and nuclear factor-kappaB (NF-κB) expressions in VSMCs (Selvendiran et al., 2009). Therefore, HO-3867, a potential derivative of curcumin, is capable of preventing vascular abnormalities by upregulating PTEN. ET-1 is known to participate in the etiologies of vascular pathologies, including hypertensive vascular remodeling, via hyperactivation of AKT and extracellular signal-regulated kinase 1/2 (ERK1/2) signaling. Curcumin treatment prevents ET-1-induced activation of AKT, ERK1/2, c-Raf, insulin-like growth factor type 1 receptor (IGF-1R), and early growth response (Egr)-1, which are well-known mitogenic and proliferative signaling molecules in VSMCs (Kapakos et al., 2012). Ang II is implicated in the proliferation and migration of VSMCs, contributing to the development and progression of vascular disorders, including atherosclerosis and hypertension. Mounting evidence indicates that activation of peroxisome proliferator-activated receptor-γ (PPAR-γ) circumvents Ang II-induced inflammation response and ROS production in VSMCs. This drives a possibility that curcumin might protect against Ang II-induced oxidative stress and inflammatory responses in VSMCs by upregulating PPAR-γ. Indeed, a study by Li et al. (2017) has shown that curcumin attenuates Ang II-induced expressions of inflammatory factors and oxidative stress through induction of PPAR-γ in VSMCs, which is accompanied by suppression of VSMC proliferation. In keeping with this, nicotinate-curcumin, an esterification derivative of niacin and curcumin, markedly prevents Ang II-induced VSMC phenotype switching, proliferation, and migration via regulating the PTEN/AKT pathway (Sun et al., 2021). As a group of short non-coding RNA, micro RNAs (miRNA) plays a critical role in the process of human diseases, including hypertension (Zhang and Sun, 2020; Zhang and Sun, 2021). Studies have illustrated that miR-22 plays an important role in vascular remodeling (Zheng and Xu, 2014), cardiac hypertrophy (Gurha et al., 2012; Huang et al., 2013), and spontaneous hypertension (Friese et al., 2013). It remains unknown as to whether curcumin regulates VSMC phenotype and proliferation by regulating miR-22. As expected, curcumin upregulated the expression of miR-22 to decrease the protein expression of specificity protein 1 (SP1), leading to an obvious inhibition of VSMC proliferation and migration, as well as vascular neointimal hyperplasia after vascular injury (Zhang et al., 2020). Chemerin, a novel adipokine, plays a crucial role in the process of atherosclerosis by acting on chemokine-like receptor 1 (CMKLR1) (Liu et al., 2019), and the chemerin/CMKLR1 signaling pathway is also involved in the regulation of intimal hyperplasia and hypertension (Kennedy et al., 2016; Artiach et al., 2018). A recent study has demonstrated that knockdown of CMKLR1 markedly inhibits VSMC proliferation and migration, and the lipocalin-2 (LCN2) acts as a key factor to mediate CMKLR1-induced VSMC functions through the p38 MAPK and Wnt/β-catenin signaling pathways (He et al., 2021). Importantly, curcumin targets this complex axis to inhibit VSMC proliferation and migration, thereby reducing atherosclerotic progression (He et al., 2021). Totally, curcumin and its analogues may be of potential use in the prevention or treatment of vascular diseases, including hypertensive vascular remodeling.

Induction of HO-1 is documented to obliterate the proliferation of VSMCs by upregulation of the cyclin-dependent kinase inhibitor p21, a negative dominator in cellular proliferation (Lee et al., 2004; Kim et al., 2009). A study by Shyy’s group has illustrated that the phytochemical curcumin increments HO-1 expression through the nuclear translocation of Nrf2, a pivotal event involved in the suppression of VSMC proliferation (Pae et al., 2007). Additionally, curcumin inhibits the growth of TNF-α-induced human VSMCs in a HO-1-dependent manner since HO-1 inhibitor tin protoporphyrin abolishes the effects of curcumin in the context of TNF-α (Pae et al., 2007). Oxidized low-density lipoprotein (ox-LDL) is progressively increased in atherosclerosis, and it causes damages in blood vessels by inducing the conversion of a normal VSMC contraction phenotype to an abnormal synthetic phenotype, resulting in the migration of VSMCs into the intima (Allahverdian et al., 2014; Di and Maiseyeu, 2021). The protective effects of curcumin on ox-LDL-induced VSMC injury were examined by an *in vitro* experiment that VSMCs were treated with ox-LDL in the presence or absence of curcumin (Wang G. et al., 2021). The authors found that curcumin-mediated photodynamic therapy alleviated the phenotypic transformation, proliferation, and migration of VSMCs upon ox-LDL exposure, an effect that is associated with induction of autophagy (Wang G. et al., 2021). Furthermore, we have also disclosed that curcumin serves as an inhibitor of histone acetyltransferases (HAT) to suppress NF-κB upregulation and nod-like receptor family protein 3 (NLRP3) inflammasome activation in VSMCs, thus preventing VSMC phenotype switching, proliferation, migration, and vascular remodeling in SHR (Sun H.-J. et al., 2017; Han et al., 2019). We further found that intragastric administration of curcumin attenuated hypertension, repressed NF-κB activation, NLRP3 and MMP-9 expressions in the aortas, reduced the media thickness and the ratio of media thickness to lumen diameter in the aortas of SHR (Han et al., 2019). Extracellular vesicles are becoming a research hotspot since they are recognized as potential therapeutic targets and drug delivery systems by transferring their loaded molecules such as miRNA, proteins, and cytokines between cells (Sun HJ. et al., 2017). Correspondingly, extracellular vesicles-mediated interactions between vascular endothelial cells and VSMCs are essential in the development of cardiovascular diseases, including hypertension (Zhang and Sun, 2022). Treatment with normal endothelial extracellular vesicles reduces the proliferation and migration of VSMCs, whereas treatment with LPS-induced endothelial extracellular vesicles promotes the proliferation of VSMCs by regulating several miRNAs, including miR-92a-3p, miR-126-5p, miR-125a-3p, miR-143-3p, to name a few (Xiang et al., 2021). Intriguingly, the same group further showed that treatment with curcumin and nicotinic-curcumin reduced endothelial extracellular vesicle secretion, possibly by inhibiting inflammation (Xiang et al., 2021), indicating that curcumin might regulate vascular functions through extracellular vesicles-mediated vascular cell communications in hypertension. This hypothesis still awaits further research.

The evidence for the favorable role of curcumin in PAH-related vascular remodeling is emerging. The thickness of pulmonary arterial media smooth cell layer and collagen fibers in adventitia tend to be normal in hypoxic hypercapnic rats treated with curcumin, thus leading to a decrease in pulmonary arterial pressure, in conjunction with a reversal of pulmonary vessel remodeling (Lin et al., 2006). Likewise, curcumin exposure promotes pulmonary VSMC apoptosis by regulating mitochondrial function, and prevents the expressions of pro-proliferative genes in pulmonary VSMCs by suppressing the PI3K/AKT pathway (Chen et al., 2021). Collectively, we and other groups provide direct evidence that curcumin plays a beneficial role in antagonizing vascular inflammation and remodeling in hypertension. However, whether curcumin could be used as an adjuvant or supplement for hypertension treatments in clinical applications still requires more verification and corroboration. Thoracic aortic aneurysm and abdominal aortic aneurysm are prevalent aortic disorders, which are characterized by VSMC phenotypic switching, VSMC apoptosis, extracellular matrix degradation, increased matrix metalloproteinase activity, secretion of inflammatory cytokines, and oxidative stress (Lu and Du, 2021). Preclinical studies have found that administration of curcumin is effective in suppressing the development of experimental aortic aneurysm by inhibiting VSMC phenotypic switching and apoptosis (Fan et al., 2012; Hosseini et al., 2021). However, these experiment results could not be reproduced in human studies. A parallel-group, randomized, placebo-controlled trial revealed that perioperative oral curcumin had no beneficial effects in elective abdominal aortic aneurysm repair, and induced a higher risk of acute kidney injury in 606 patients (Garg et al., 2018). In spite of this inconsistence, more preclinical and clinical studies are required to confirm the potential benefits of curcumin and its analogs in aortic aneurysm.

## Amelioration of Endothelial Cell Dysfunction

As a large paracrine organ, the vascular endothelium plays an important role in the regulation of cell growth, vascular tone, platelet and leukocyte interactions, as well as thrombogenicity (Sun et al., 2016; Gong et al., 2019). Disruption of endothelial function is a pivotal event in initiating various disorders, including arterial hypertension and PAH (Monteiro et al., 2019; Zhang and Sun, 2020). Endothelial dysfunction is hallmarked by increased ROS, overproduction of proinflammatory factors, deficiency of NO bioavailability, imbalanced productions in endothelium-released relaxing and contracting factors, enhanced leukocytes adhesion and permeability of endothelium (Monteiro et al., 2019). Furthermore, the proliferation, migration, and apoptosis of endothelial cells are intimately linked with endothelial dysfunction in hypertension and PAH (Zhang et al., 2018). Therapeutic strategies against vascular endothelial dysfunction are essential for preventing and treating vascular lesions, such as hypertension. A review has summarized that curcumin could improve endothelial dysfunction through its anti-inflammatory, anti-aging, antiangiogenic, and antioxidant properties (Zhang et al., 2018), suggesting that curcumin might ameliorate hypertension and its associated cardiovascular remodeling by reversing endothelial damage (Alidadi et al., 2021).

Endothelium-dependent vasorelaxation is impaired in aortic rings isolated from 2K1C hypertensive rats, which is compromised by curcumin treatment (Boonla et al., 2014). Additionally, administration of curcumin reverses hypertension-induced oxidative stress, vascular structural modifications, and eNOS inactivation (Boonla et al., 2014). Preservation of endothelial function is sufficient for curcumin to reduce blood pressure and decrease hindlimb vascular resistance (Boonla et al., 2014). As mentioned above, COX-2 is not only a driver for the exaggerated vasoconstrictor, but also a stimulator for vasodilation dysfunction in hypertension. Demethoxycurcumin rescued the attenuated endothelium-dependent relaxations, which was accompanied by the normalization of COX-2 expression in the renal arteries of SHR (Li and Tian, 2016). Tetrahydrocurcumin is established to prevent the elevation of blood pressure, peripheral vascular resistance, and aortic stiffness in rats after L-NAME administration (Nakmareong et al., 2012). These changes are associated with increased aortic eNOS expression, elevated plasma nitrate/nitrite, decreased oxidative stress, and enhanced blood glutathione (Nakmareong et al., 2012). Induction of HO-1 by curcumin alleviated metabolic syndrome-related hypertension and vascular complications by maintaining endothelial-dependent relaxation and NO generation in blood vessels (El-Bassossy et al., 2014). Curcumin is identified to be major constituents in turmeric and black seeds, whilst co-administration of black seeds and turmeric lowers blood pressure and hypertriglyceridemia, hyperinsulinemia, and endothelial dysfunction in fructose-fed rat model of metabolic syndrome (Amin et al., 2015). In chronic cadmium exposure, curcumin and tetrahydrocurcumin protect vascular endothelium by increasing NO bioavailability and improving vascular function, thereby relieving vascular dysfunction and high blood pressure caused by cadmium toxicity (Kukongviriyapan et al., 2016; Almenara et al., 2020). In line with this, supplementation with curcumin significantly reduces blood pressure, alleviates oxidative stress, increases plasma nitrate/nitrite and glutathione in rats with chronic exposure to lead and cadmium (Tubsakul et al., 2021). These beneficial effects of curcumin are associated with the upregulation of the eNOS and subsequent improvement of vascular responsiveness (Tubsakul et al., 2021). Elevated blood pressure, increased oxidative stress, decreased plasma NO levels, and downregulation of eNOS expression in aortic tissues are found in L-NAME-induced hypertensive rats, whereas this phenomenon is largely eradicated by hexahydrocurcumin, a major metabolite of curcumin (Panthiya et al., 2022).

Injection of iron sucrose (10 mg/kg/day) for 8 weeks in male ICR mice induces iron overload, hypertension, impaired vascular function and blunted response of the autonomic nervous system (Sangartit et al., 2016). These abnormalities are corrected by tetrahydrocurcumin in combination with deferiprone (Sangartit et al., 2016). Electron microscope showed that the endothelial cells of pulmonary arterioles tend to be normal after curcumin treatment in PAH rats (Li et al., 2014). A clinical study has found that supplementation of curcumin improves resistance artery endothelial function in healthy middle-aged and older adults by increasing vascular NO bioavailability and reducing oxidative stress (Santos-Parker et al., 2017), further confirming the benefits of curcumin in vascular endothelial function. Overall, pharmacological intervention of endothelial dysfunction by curcumin could be utilized as an effective avenue for hypertension therapy. These above findings hint that curcumin grants protection against hypertension due to its benefits on the endothelium. It is anticipated that the continuous studies of curcumin in the vascular endothelium will provide great hope for therapeutic approaches for hypertension.

The endothelial to mesenchymal transition (EndMT) has recently emerged as one of the key phenomena driving endothelial dysfunction and vascular remodeling in several vascular diseases, such as arterial hypertension and PAH (Li et al., 2018; Botts et al., 2021). Consequently, inhibition of EndMT might provide novel therapeutic approaches for vascular inflammation-related diseases (Lu et al., 2019; Xu et al., 2021). Chen and coworkers found that curcumin inhibited transforming growth factor β1 (TGF-β1)-induced EndMT in endothelial cells Nrf2-upregulated dimethylarginine dimethylaminohydrolase-1 (DDAH1) expressions, leading to a reduction in endothelial cell fibrosis (Chen et al., 2020). However, there is no direct evidence showing that curcumin attenuated the development of hypertension by restraining the process of EndMT in endothelial cells. It is certain that the regulation of EndMT by curcumin could provide novel insights into hypertension-induced endothelial dysfunction, thus yielding novel therapeutic approaches.

## Blockade of the Renin Angiotensin System

It is well known that activation of the RAS plays a pathogenic role in the development and progression of hypertension. As a principal effector peptide within RAS, Ang II is a potential regulator of arterial blood pressure by binding to two distinct receptors: the AT1R and AT2R. Most actions of Ang II are primarily transmitted via AT1R, such as vasoconstriction, cardiac contractility, and reduced vascular compliance. Blockade of AT1R is an efficient approach to attenuate blood pressure in hypertensive patients and animals. Yang et al. have found that curcumin dose- and time-dependently downregulates AT1R expressions in VSMCs through reducing the binding of SP1 with the AT1R promoter, suggesting that the effect of curcumin on AT1R expression at the transcriptional level (Yao et al., 2016). In addition, these authors further demonstrate that curcumin treatment reduces Ang II-induced hypertension and vasoconstriction, concomitant with reduction of AT1R expression in the arteries, indicating that downregulation of AT1R is an important mechanism for curcumin to prevent the development of hypertension in an Ang II-induced hypertensive models (Yao et al., 2016).

Angiotensin-converting enzyme (ACE), a membrane-bound enzyme, is known to act on diverse peptide substrates in the extracellular space, and this peptidyl dipeptidase cleaves off His-Leu from angiotensin I to produce Ang II (identical to kininase II), which cleaves off Phe-Arg from bradykinin to yield inactive residue (Gouda et al., 2021). Thus, inhibition of ACE is capable of blocking the formation of Ang II and potentiating the effects of bradykinin (Marceau and Bachelard, 2020). A large body of studies are devoted to demonstrate the therapeutic potential of ACE inhibitors for hypertension (Prieto et al., 2021). A series of experiments have shown that the actions of bradykinin are mainly mediated by its receptors B1 and B2 (Cui et al., 2005). Incubation of Ang II stimulates gene expression of both B1 and B2 receptors in cardiomyocytes and VSMCs, an effect that is abolished by concurrent inhibition of the AT1 receptor with losartan (Kintsurashvili et al., 2001). Similarly, exogenous ACE strongly upregulates the genes of bradykinin receptors B1 and B2 in VSMCs, whereas this phenomenon is not altered by addition of specific Ang II antagonists for the AT1 and AT2 receptors, as well as the ACE inhibitor captopril (Kintsurashvili et al., 2001). It is reported that curcumin could suppress the formation and action of NF-κB and AP-1 through interacting with such transcriptional factors (Aggarwal et al., 2003; Kang et al., 2004). Interestingly, pretreatment with transcriptional inhibitor curcumin completely abolishes the effects of ACE on B1 and B2 receptors, suggesting that ACE challenge results in upregulations of the bradykinin B1 and B2 receptor genes through activating the NF-κB and AP-1 signaling pathways (Kintsurashvili et al., 2001). As a widely used compound, curcumin might be used to treat hypertension by modulating the interaction of ACE with bradykinin system in VSMCs dependent on NF-κB and AP-1 signaling.

## Other Mechanisms

Oral gavage of curcumin partially prevents L-NAME-induced hypertension in rats, accompanied by decreased wall thickness and cross-sectional area of the aorta (Hlavačková et al., 2011), indicating that administration of curcumin is effective in preventing negative changes in blood vessel morphology under hypertensive conditions. Interestingly, it is reported that tetrahydrocurcumin, a major metabolite of curcumin, could inhibit the elevation of blood pressure, restore vascular responsiveness to vasoactive substance, and suppress vascular resistance of rats treated with L-NAME, an effect that is likely to be more effective than those of curcumin (Nakmareong et al., 2011). Mechanistically, the improvement of hypertensive vascular remodeling by tetrahydrocurcumin is mediated by suppression of oxidative stress and nitrative stress in the aortic tissues (Nakmareong et al., 2011). Moreover, Saowanee and coworkers also demonstrated that tetrahydrocurcumin treatment reversed the deleterious effects of L-NAME on blood pressure, peripheral vascular resistance, aortic stiffness, and oxidative stress in rats (Nakmareong et al., 2011). The favorable actions of tetrahydrocurcumin were related to increased aortic eNOS expression, elevated plasma nitrate/nitrite, decreased oxidative stress with reduced superoxide production and enhanced blood glutathione (Nakmareong et al., 2011). In parallel to this, L-NAME supplementation leads to upregulations of NF-кB, vascular cell adhesion molecule 1 (VCAM-1), intercellular adhesion molecule 1 (ICAM-1), TNF-α, p-ERK1/2, phosphorylated-c-Jun N-terminal kinases (p-JNK), phosphorylated-mitogen activated protein kinase p38 (p-p38), transforming growth factor-beta 1 (TGF-β1), MMP-9 and collagen type 1 in rat aortas, with a concomitant decrease in eNOS expression in aortic tissues (Panthiya et al., 2022). However, these abnormal alterations are obviously reversed by hexahydrocurcumin treatment (Panthiya et al., 2022). Growing evidence has highlighted the importance of gut dysbiosis and gut-brain communication dysregulation in the pathogenesis of hypertension (Li et al., 2020; Sharma et al., 2020). Transplantation of fecal bacteria from normotensive rats to hypertensive rats is found to alleviate the development of hypertension (Toral et al., 2019). Retrograde viral tracing in hypertensive rodents showed increased neural connections from the intestine to the hypothalamus paraventricular nucleus (PVN), an important cardioregulatory region in the central nervous system (Toral et al., 2019). This provides intuitive evidence to recognize the microbiota-gut-brain axis as a new mechanism involved in hypertension pathologies (Toral et al., 2019). As anticipated, the elevated blood pressure of SHR is markedly inhibited by curcumin treatment by altering the gut microbial composition and improving intestinal pathology and integrity (Li et al., 2021). The gut benefits of curcumin are related to reduced neuroinflammation and oxidative stress in the PVN (Li et al., 2021). This observation suggests that reconstruction of the gut microbiota and normalization of the gut-brain communications partially contribute to the anti-hypertensive effects of curcumin. Previously, the dietary compound curcumin has been documented to prevent heart failure-induced increases in both myocardial wall thickness and diameter by inhibiting p300-HAT activity (Morimoto et al., 2008). Very recently, the same research group has shown that oral supplementation of curcumin decreases hypertension-induced increase in posterior wall thickness and left ventricle mass index, even though without affecting blood pressure and systolic function in Dahl salt-sensitive rats (Sunagawa et al., 2021). Mechanistically, curcumin acts as an inhibitor of p300-HAT activity to attenuate the acetylation levels of GATA binding protein 4 (GATA4), a hypertrophy-responsive transcription factor in the hearts of hypertensive rats (Sunagawa et al., 2021).

**FIGURE 3:**
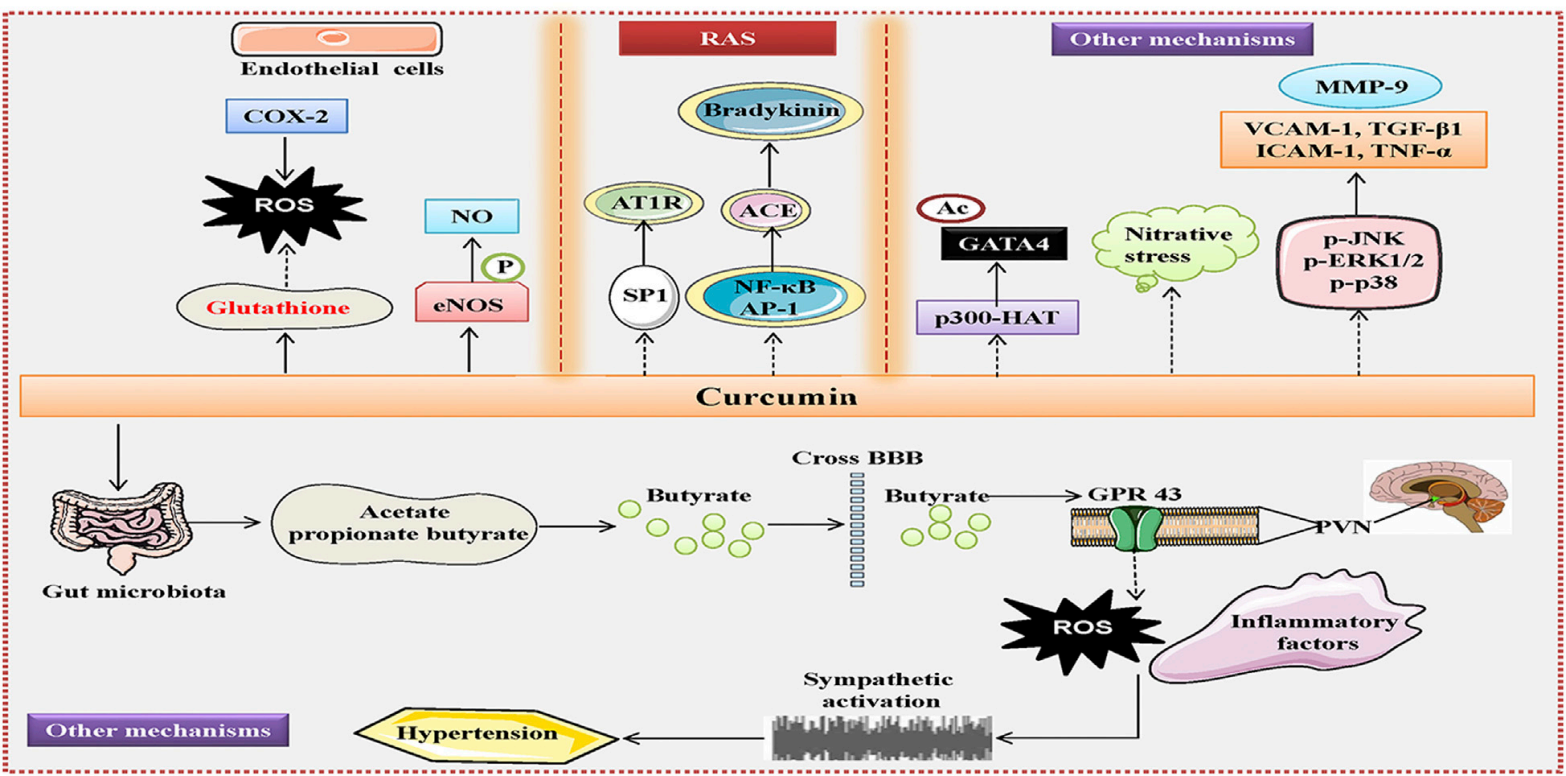
Curcumin-mediated anti-hypertensive signaling pathways, associated with amelioration of endothelial dysfunction and inhibition of the RAS, as well as other mechanisms. Curcumin attenuates the development of hypertension and vascular remodeling through increasing NO bioavailability, decreasing oxidative stress, nitrative stress, and inflammation in the endothelium, blocking of the RAS, inhibiting the p300-HAT to reduce GATA4 acetylation, altering the gut microbial composition and improving intestinal pathology and integrity, inhibiting neuroinflammation and oxidative stress in the PVN. NO, nitric oxide; RAS, renin angiotensin system; HAT, histone acetyltransferase; GATA4, GATA binding protein 4; GPR 43, G protein-coupled receptor 43; PVN, paraventricular nucleus.

These published results suggest that curcumin and its metabolites may be used as dietary supplements to antagonize hypertension and its associated vascular dysfunction through suppressing vascular inflammation, oxidative stress, restoring eNOS/NO signaling, reshaping gut microbial composition, and inhibiting p300-HAT activity (Figure 3). Despite this, further studies are required to determine whether tetrahydrocurcumin is superior to curcumin in the treatment of hypertension and vascular remodeling and to explore the underlying mechanisms. Although both women and men develop high blood pressure, the sex differences in hypertensive prevalence are well recognized for decades (Tipton and Sullivan, 2014; Muiesan et al., 2016; Ramirez and Sullivan, 2018; Zhang and Sun, 2020). In other words, men have a higher prevalence of hypertension than women before menopause, whereas a higher prevalence of hypertension is found in women after menopause relative to age-matched men (Muiesan et al., 2016), indicating a regulator role of estrogen in regulating blood pressure. Despite the sex differences in the prevalence of hypertension, the current treatment guidelines appear to be the same for different genders (James et al., 2014). To date, no studies have been carried out to compare the therapeutic effect of curcumin on hypertension in different genders. Considering the continuous advancement of individualized treatment in hypertension, it will be interesting to know whether the blood pressure lowering actions of curcumin are related to gender differences.

## Clinical Trials of Curcumin

The healthy benefits of curcumin in hypertension have gained tremendous attention because of the increasing epidemic of hypertension. However, these preclinical results await further clinical translation. Actually, the clinical trials of curcumin have been flourishing during the last decades. Acute coronary syndrome (ACS), a pathological condition whereby the blood supply to the cardiac tissues is cut off, is composed of ST-segment elevation myocardial infarction, non-ST-segment elevation myocardial infarction, and unstable angina (Paradiso-Hardy et al., 2003). Dyslipidemia and hyperglycemia are characteristic features in patients with ACS (Goyal et al., 2004). A randomized, double-blind, controlled trial has shown that oral administration of curcumin for 2 months obviously reduces total cholesterol and low-density lipoprotein cholesterol in ACS patients (Alwi et al., 2008). This study suggests the lipid-lowering effects of curcumin in subjects suffering from ACS. In similarity with this study, administration of curcumin for 7 days effectively reduces serum total cholesterol levels by 11.63%, and increased serum HDL cholesterol by 29% in ten healthy human volunteers (Soni and Kuttan, 1992), indicating that curcumin might act as a chemopreventive agent against diseases related to lipid metabolism disturbance, such as atherosclerosis. However, improving the lipid profiles does not mean that curcumin is protective against ACS and/or atherosclerosis. Further research is warranted to examine whether curcumin could delay or reverse the development of ACS and/or atherosclerosis.

The blood glucose-lowering effects of curcumin were first reported in 1972 (Srinivasan, 1972), and Srinivasan found that ingestion of turmeric powder (5 g) over a period reduced the blood sugar level from 140 to 70 mg/dl in a male patient who had diabetes for 16 years (Srinivasan, 1972), suggesting curcumin’s ability to decrease blood glucose levels. One study evaluated the effects of curcuminoids (NCB-02) in reducing oxidative stress and inflammatory markers in type 2 diabetic patients (Usharani et al., 2008). Intake of NCB-02 (300 mg of curcumin, twice a day) significantly improved endothelial function and reduced oxidative stress and inflammatory markers in these patients with type 2 diabetes (Usharani et al., 2008). (Wickenberg et al., 2010) assessed the actions of curcuma longa on postprandial plasma glucose, insulin levels and glycemic index in healthy subjects, and they found that ingestion of curcuma longa increased postprandial serum insulin levels, without affecting plasma glucose levels and glycemic index in healthy participants (Wickenberg et al., 2010). This study suggests the capability of curcuma longa to promote insulin secretion. Another group conducted a randomized, double-blinded, placebo-controlled trial to examine the effects of curcumin in delaying development of type 2 diabetes mellitus in the prediabetic population (Chuengsamarn et al., 2012). They found that after 9 months of treatment, curcumin-treated patients exhibited a better overall function of β-cells, higher pancreatic β cell function (HOMA-β) and adiponectin, lower C-peptide and Homeostatic Model Assessment for Insulin Resistance (HOMA-IR) when compared with the placebo group in prediabetic subjects (Chuengsamarn et al., 2012). This study demonstrated for the first time that curcumin intervention may be beneficial for a prediabetic population. However, a study by Hodaei et al. found that supplementation with curcumin caused a beneficial effect on the body weight, body mass index, waist circumference, and fasting blood glucose, but had no effect on the hemoglobin A1c (HbA1c), insulin, malondialdehyde (MDA), total antioxidant capacity (TAC), HOMA-IR, and HOMA-β in overweight patients with type 2 (Hodaei et al., 2019). Regarding these discrepancies, long-term trials with larger number of patients are needed to confirm whether curcumin’s effects on diabetes are transient or long-lasting.

Diabetic kidney disease is believed to be a major cause of chronic kidney disease and end stage kidney disease (ESKD), which is associated with high mortality and morbidity worldwide (Sun H. J. et al., 2019). A previous study was conducted to investigate the effects of curcumin on serum and urinary levels of TGF-β, IL-8, and TNF-α, as well as proteinuria, in patients with diabetic nephropathy (Khajehdehi et al., 2011). This study demonstrated for the first time that serum concentrations of TGF-β and IL-8, as well as urinary protein excretion were significantly decreased in these patients after turmeric supplementation (Khajehdehi et al., 2011). Most importantly, no adverse effects were detected during the supplementation of turmeric, indicating that turmeric could be recommended as a safe adjuvant therapy for patients with diabetic nephropathy. Despite this, larger, randomized clinical trials should further confirm the above observations, and determine the optimal concentration and duration of curcumin to achieve the therapeutic effects.

Likewise, curcumin supplementation may exert beneficial effects on the management of hypertension. A double-blinded, randomized, controlled trial was implemented to explore whether an enhanced bioavailable curcumin formulation, CurQfen^®^, could improve cardiovascular disease-related blood biomarkers and arterial function in young obese men (Campbell et al., 2019). They showed that intervention of curcumin for 12 weeks obviously lowers the levels of homocysteine, and enhances the levels of high-density lipoprotein levels in these young obese subjects when compared to the placebo group (Campbell et al., 2019). However, no changes in endothelial function, augmentation index, central blood pressure, the circulating levels of glucose, insulin, leptin, and adiponectin were observed in the curcumin group relative to the control group (Campbell et al., 2019). Morand’s group examined the effects of an acute intake of curcumin on vascular functions in 18 healthy smokers, and their results showed that intake of curcumin for 2 h had no significant effect on vascular function assessed by flow-mediated dilation (Barber-Chamoux et al., 2018). A subgroup analysis on the basis of the gender or the cardiovascular-risk score revealed that curcumin exerted an obvious effect on vascular function in both women and subjects with lower cardiovascular risk (Barber-Chamoux et al., 2018). This clinical trial highlights a huge variability in the efficacy of curcumin across smokers, which may be mainly due to gender differences and cardiovascular risk levels (Barber-Chamoux et al., 2018). A clinical trial results showed that curcumin supplementation for 12 weeks improves resistance artery endothelial function by increasing vascular NO bioavailability and reducing oxidative stress in thirty-nine healthy middle-aged and older adults (Santos-Parker et al., 2017). A randomized and placebo-controlled study was carried out to determine the actions of curcumin on the clinical symptoms in patients suffering from relapsing or refractory lupus nephritis (Khajehdehi et al., 2012). This study showed that oral supplementation of turmeric was effective in ameliorating proteinuria, hematuria, and systolic blood pressure in such patients, representing turmeric as an adjuvant safe therapy for subjects with lupus nephritis (Khajehdehi et al., 2012). Although a wide range of clinical trials on curcumin’s benefits in human ailments have been completed, the exact roles of curcumin in arterial hypertension and PAH are needed to evaluated by long-term clinical studies with large samples. A search on www.clinicaltrials.gov (accessed in December 2021) indicated that a total of 210 clinical trials were conducted to examine the role of curcumin in different human diseases, and further analysis revealed that 39 clinical trials with curcumin are ongoing. These currently progressing clinical studies provide robust evidence to confirm curcumin’s therapeutic potential for human diseases. Interestingly, almost the majority of clinical trials are investigating curcumin’s therapeutic effects on human diseases, rather than its metabolites or analogs, although the metabolites or analogs of curcumin might hold better benefits on human health. More clinical trials are still needed to verify the pros and cons of between curcumin and its metabolites/analogs in the treatment of human diseases, thus maximizing the clinical benefits of curcumin or its metabolites/analogs. Here, we also hope that there will be more clinical trials to explore and verify the antihypertensive effects of curcumin and its analogs.

## Challenges and Future Directions

As a naturally occurring bioactive compound, curcumin plays a critical role in human nutrition because of its anti-oxidative and anti-inflammatory abilities. As such, curcumin is known as a promising candidate that can prevent and treat a wide spectrum of diseases. Indeed, the evidence for the potential of curcumin as a therapeutic agent and/or nutraceutical has greatly increased during the last several decades. The increased interest has spurred the growth of preclinical and clinical studies to assess the efficiency of curcumin and its formulations. Although the great therapeutic potential of curcumin in various diseases, including arterial hypertension and PAH, these findings should be treated carefully before clinical translation due to some crucial challenges.

Firstly, the poor bioavailability of curcumin leads to inconsistent and unstable results, and this restricts the therapeutic usages of curcumin in functional and nutritious foods. Secondly, the lower plasma and tissue levels of curcumin may be attributed to its poor absorption, rapid metabolism, and fast elimination, resulting in a low level of free curcumin even if a high concentration of curcumin is used (Kunati et al., 2018). This results in a great possibility that the metabolites of curcumin may be responsible for the biological and pharmacological actions rather than the free curcumin (Nelson et al., 2017). Once absorbed in the body, curcumin may undergo the modification of conjugations, such as sulfation and glucuronidation, and the major metabolites of curcumin are glucuronides of tetrahydrocurcumin and hexahydrocurcumin (Anand et al., 2007). In addition, dihydroferulic acid, ferulic acid, and sulfate conjugates are minorbiliary metabolites of curcumin (Anand et al., 2007). Intriguingly, tetrahydrocurcumin and hexahydrocurcumin, known as curcumin metabolites, are found to hold considerable promise in the treatment of various diseases, including hypertension. Thus, it is highly possible that the metabolites of curcumin might account for its antihypertensive effects, which merits further studies. Thirdly, the concentrations of curcumin vary in different foods or plants, the optimal dosage levels of curcumin with therapeutic effects are difficult to determine. Fourthly, different experimental conditions might be responsible for a big gap between animal and human studies. Of note, the method of extraction of the bioactive ingredients in curcumin may be an important factor affecting its blood pressure lowering effects. Further research is required to determine which bio-extraction method would yield curcumin with the highest bioactivity and the lowest toxicity. It is quite difficult to directly use curcumin-derived treatment without enough clinical studies even though animal studies are available. Fifthly, animal studies are disputable since the different dose levels, research conditions, treatment time, and route of administration are used in distinct studies. Sixthly, in addition to the benefits of curcumin, we should also pay more attention to its side effects during its usages in preclinical and clinical studies. A study by Fu et al. examined the toxicity of liposomal curcumin in animal models, and results showed that dose-dependent hemolysis occurred when the therapeutic dose exceeded 20 mg/kg (Fu et al., 2021). A small number of diabetic patients experienced side effects, such as constipation and nausea, after oral administration of curcumin at a dose of 1.5 g/kg for 6 months (Patel et al., 2020). Lao et al. (2006) determined the maximum tolerable dose and safety of curcumin after a single oral dose (500–12,000 mg) in 34 healthy volunteers, and they found that 7 participants experienced diarrhea, headache, rash, and yellow stool. A phase I clinical trial explored the pharmacology of curcumin in fifteen patients with advanced colorectal cancer, and curcumin at doses ranging from 0.45 to 3.6 g/day for 1–4 months increased serum alkaline phosphatase and lactate dehydrogenase contents, along with nausea and diarrhea (Sharma et al., 2004). These studies might provide possible evidence that curcumin might cause several side effects at high concentrations which is a basis for some concern. More work is necessary to establish safe effective dose levels of curcumin. Similar to conventional drugs, curcumin is capable of drug-drug interactions with other medicines, which may result in lower effectiveness or increased toxicity (Bahramsoltani et al., 2017). For example, co-administration of curcumin with a number of conventional pharmacological drugs induced pharmacokinetic changes, including changes in maximal plasma concentration (Cmax) and area under the concentration time curve (AUC) (Bahramsoltani et al., 2017). Inhibition of cytochrome isoenzymes and P-glycoprotein may contribute to such drug interactions (Patel et al., 2020). Taking increased amounts of curcumin might also inhibit platelet aggregation and increase the activity of liver enzymes, induce gastrointestinal disorders, or contact dermatitis and hives (Fadus et al., 2017). However, the evidence for the curcumin-drug interactions is still lacking. Since then, more *in vitro* and *in vivo* studies are encouraged to judge the drug interactions of curcumin, thus avoiding unnecessary side effects when curcumin was used concomitantly with conventional pharmacological drugs. Dose-escalating results from clinical studies suggest that the safety of curcumin at doses up to 12 g/day over 3 months (Gupta et al., 2013). However, the clinical benefits of curcumin did not appear to be dose-related (Gupta et al., 2013). The dose-response effects of curcumin on mitigating vascular inflammation should be therefore carefully determined in order to establish the most effective dose of curcumin without toxic side effects. Overall, determining the most effective dose of curcumin without toxic side effects might help to recommend this fascinating polyphenol at the fore front of novel therapeutics. Seventhly, most of the animal experiments are conducted to determine whether curcumin could prevent hypertension since curcumin is mostly administered before or a few days before the hypertensive model starts, indicating a preventive aspect of curcumin in hypertension. Thus, more research is needed in the future to confirm whether curcumin is able to treat hypertension, i.e., curcumin is given at the middle and late stages of hypertensive models to confirm the curative effects of curcumin on hypertension, not just its preventive effects. Last but not least, it is still a challenge to recognize curcumin and its derivatives as prebiotics in the human gut although they possess the ability to reshape the composition of the gut microbiota. It is deserved to investigate which probiotics could be affected by curcumin, not only in hypertension, and it is also worth studying whether supplementation of probiotics into curcumin formulations could synergistically improve the efficiency of curcumin. It is hopeful that further research is needed to unravel these limitations and improve the efficacy of curcumin against hypertension.

In order to solve these above limitations, scientists have made great efforts. For instance, different modifications of curcumin and its delivery systems are developed to increase the stability, solubility, *in vivo* uptake, bioactivity, and safety of curcumin, such as nanoparticles, micellation, and conjugation with other materials (Adahoun et al., 2017; Akbar et al., 2018; Khayyal et al., 2018; Xu et al., 2018). It is certain that these modifications will greatly facilitate the development of curcumin-derived novel therapies with few side effects and high bioavailability, and improve the possibility of clinical transformation of curcumin. Given the capability of curcumin to reverse vascular remodeling in hypertension, curcumin may be postulated to be an attractive compound to prevent and treat hypertension and vascular remodeling through modulating a wide spectrum of signaling pathway. Nevertheless, the complex regulatory mechanisms of curcumin in hypertension-associated vascular remodeling are incompletely understood. As a consequence, more studies are required to understand how curcumin and its metabolites benefit vascular dysfunction induced by hypertension.

## Conclusion

Taken together, this review summarizes and discusses the therapeutic roles and molecular mechanisms of curcumin in hypertension and its related vascular remodeling (Tables 1, 2). The novel insights into hypertensive vascular remodeling are highly acquired because of the increased incidence of hypertension. Continuous *in vitro*, *in vivo*, and clinical studies have identified the underlying molecules in curcumin-mediated benefits in hypertension-related vascular remodeling. As of yet, a growing body of impressive studies focusing on the effects of curcumin and its analogues on hypertensive vascular damage are growing in recent years. Importantly, nanotechnology is used to encapsulate curcumin, thereby enhancing its stability, bioavailability, bioactivity, and health benefits. Most importantly, more efforts are warranted to eliminate the debate between preclinical and human studies of curcumin. With our in-depth understandings towards the mechanistic network of curcumin in hypertensive vascular remodeling, we anticipate multiple novel curcumin therapeutics to evolve for hypertension and its associated vascular remodeling in the near future.

**TABLE 1:** Vascular benefits of curcumin in hypertension from *in vivo* studies.

Formulations	Dose	Disease model	Benefits	References
Curcumin	50 and 100 mg/kg/d	L-NAME-induced hypertensive rats	Suppressing the blood pressure elevation; decreasing vascular resistance; restoring vascular responsiveness; reinstating eNOS protein expression in the aortic tissues; reducing vascular oxidative stress	Nakmareong et al. (2011)
Tetrahydrocurcumin	50 and 100 mg/kg/d	L-NAME-induced hypertensive rats	Suppressing the blood pressure elevation; decreasing vascular resistance; restoring vascular responsiveness; reinstating eNOS protein expression in the aortic tissues; reducing vascular oxidative stress; the antihypertensive effects of tetrahydrocurcumin are apparently more potent than curcumin	Nakmareong et al. (2011)
Tetrahydrocurcumin	50 and 100 mg/kg/d	L-NAME-induced hypertensive rats	Decreasing blood pressure, peripheral vascular resistance, aortic stiffness and oxidative stress; elevating aortic eNOS expression and plasma nitrate/nitrite	Nakmareong et al. (2012)
Hexahydrocurcumin	20, 40 or 80 mg/kg/d	L-NAME-induced hypertensive rats	Inhibiting the development of hypertension, vascular dysfunction, and remodeling; exhibiting antioxidant and anti-inflammation potential	Panthiya et al. (2022)
Curcumin	100 mg/kg/d	L-NAME-induced hypertensive rats	Decreasing hypertension, wall thickness and cross-sectional area of the aorta, as well as vascular fibrosis	Hlavačková et al. (2011)
Curcumin	50 and 100 mg/kg/d	2K1C-induced hypertensive rats	Reducing plasma angiotensin converting enzyme levels; improving endothelial dysfunction and vascular remodeling; raising nitric oxide availability; reducing oxidative stress	Boonla et al. (2014)
Curcumin	60 mg/kg/d	5/6 nephrectomized rats	Attenuating systemic and glomerular hypertension; increasing plasma creatinine and blood urea nitrogen; enhancing nuclear translocation of Nrf2	Tapia et al. (2012)
Curcumin	50 mg/kg/d	Salt-sensitive Dahl rats	Preventing the deterioration of systolic function and heart failure-induced increases in myocardial wall thickness	Morimoto et al. (2008)
Curcumin	50 mg/kg/d	Salt-sensitive Dahl rats	Decreasing posterior wall thickness and left ventricle mass index; without affecting the blood pressure and systolic function	Sunagawa et al. (2021)
Curcumin	100 mg/kg/day	Spontaneously hypertensive rats	Attenuating hypertension; reducing NFκB activation, NLRP3 and matrix metalloproteinase-9 expressions and aortic media thickness	Han et al. (2019)
Curcumin	100 mg/kg/day	Spontaneously hypertensive rats	Attenuating hypertension and vascular remodeling	Sun et al. (2017a)
Curcumin	100 or 300 mg/kg/day	Spontaneously hypertensive rats	Decreasing blood pressure, altering the gut microbial composition and improved intestinal pathology and integrity	Li et al. (2021)
Curcumin	300 mg/kg/d	Ang II-induced hypertensive mice	Reducing hypertension in C57Bl/6J mice; lowering AT1R expression in the arteries and decreasing Ang II-mediated vasoconstriction in the mesenteric artery	Yao et al. (2016)
Curcumin	150 mg/kg/d	Ang II-infused hypertensive rats	Decreasing the mean arterial blood pressure; attenuating myocardial fibrosis; reducing AT1R expression; upregulating AT2R expression	Pang et al. (2015)
Curcumin in hyaluronic acid-based nanocapsules	4.5 mg/kg/d	Hypertensive TGR(m-Ren2)27 rats	Resulting in a gradual inhibition of SBP, DBP and MAP	Czyzynska-Cichon and Janik-Hazuka, (2021)
Curcumin nanoparticles	5 mg/kg/d	Rats with diet-Induced metabolic syndrome	Normalizing blood pressure; reducing the left ventricular diastolic stiffness.	Du Preez et al. (2019)
Curcumin	100 mg/L	Cadmium-induced mice	Increasing vascular responsiveness; normalizing the blood pressure levels; upregulating eNOS protein; restoration of glutathione redox ratio and alleviation of oxidative stress	Kukongviriyapan et al. (2014)
Curcumin	100 mg/L	Lead acetate- and cadmium chloride-treated rats	Reducing blood pressure; alleviating oxidative stress; increasing plasma nitrate/nitrite and glutathione in the blood	Tubsakul et al. (2021)
Tetrahydrocurcumin	50 and 100 mg/kg/d	Cadmium-induced mice	Decreasing arterial blood pressure; restoring vascular responses to vasoactive agents; decreasing aortic stiffness and hypertrophic aortic wall remodeling as well as vascular fibrosis	Sangartit et al. (2014)
Tetrahydrocurcumin	50 mg/kg/d	Iron-overloaded mice	Attenuating hypertension, vascular dysfunction, baroreflex dysfunction, and oxidative stress	Sangartit et al. (2016)
Curcumin	50 mg/kg/d	Hypoxic hypercapnic rats	Decreasing PAH; improving pulmonary vessel remodeling; inhibiting the deposition of collagen I in pulmonary arterioles	Lin et al. (2006)
Curcumin	30 mg/kg/d	MCT-induced PAH rats	Reversing pulmonary vascular remodeling	Chen et al. (2021)
Curcumin	150 mg/kg/d	Hypoxic hypercapnic rats	Inhibiting the remodeling of pulmonary vessel; blocking proliferation of pulmonary arterial media smooth cell layer and collagen fibers in adventitia	Li et al. (2014)
Curcuminoids	4–18 μM	Isolated segments of rat pulmonary artery and aorta	Possessing vasorelaxant activity on pulmonary arteries	Kruangtip et al. (2015)
Curcumin nanoparticles	50 mg/kg/d	MCT-induced PAH rats	Reducing right ventricular wall thickness and right ventricle weight/body weight ratio; inhibiting right ventricular inflammation and fibrosis	Rice et al. (2016)
Curcumin acetate nanocrystals	2 mg/kg/d	MCT-induced PAH rats	Inhibiting the development of PAH.	Hu et al. (2017)

**TABLE 2:** Vascular benefits of curcumin in hypertension from *in vitro* studies.

Formulations	Dose	Cell types	Benefits	Ref
Curcumin	20 μM	Primary VSMCs	Attenuating VSMC migration; inhibiting NLRP3 expression and IL-1β concentration in VSMCs	Han et al. (2019)
Curcumin	20 μM	Primary VSMCs	Preventing the NLRP3 inflammasome activation, VSMC phenotype switching and proliferation	Sun et al. (2017a)
Curcumin	10^–6^ M	A10 cells	Decreasing AT1R expression in a concentration- and time-dependent manner	Yao et al. (2016)
Curcumin	10 and 20 μM	Human aortic smooth muscle cells	Inhibiting TNF-α-induced migration of human aortic smooth muscle cells	Yu and Lin, (2010)
Curcumin	10^–6 ^–10^–4^ M	Rabbit VSMCs	Exhibiting a greater inhibitory effect on platelet-derived growth factor- and serum-stimulated proliferation of rabbit VSMCs	Huang et al. (1992)
Curcumin	10^–6 ^–10^–5^ M	A7R5 cells	Inhibiting the proliferation of A7R5 cells in a concentration-dependent manner	Chen and Huang, (1998)
Demethoxycurcumin	9, 18, 36, 72, or 144 μM	Primary rat VSMCs	Inhibit the migration of VSMCs by reducing the expression of MMP-2 and MMP-9	Sheu et al. (2013)
Curcumin-eluting PLLA films	0.1 mg	Human coronary artery smooth muscle cells	Preventing cell proliferation through the protein kinase (PK) and mitogen-activated protein kinase (MAPK) pathways	Nguyen et al. (2004)
Curcumin	1–25 μM	Primary rat VSMCs	Inhibiting platelet-derived growth factor-stimulated VSMC proliferation and migration	Yang et al. (2006)
Dehydrozingerone	1–50 μM	Primary rat VSMCs	Eliciting a concentration-dependent inhibition of PDGF-stimulated VSMC migration, proliferation, collagen synthesis	Liu et al. (2008)
Bisdemethoxycurcumin	5, 10, 25 μM	Primary rat VSMCs	Inhibiting PDGF-induced vascular smooth muscle cell motility and proliferation	Hua et al. (2013)
Curcumin	1, 10, and 100 µM	Rat VSMCs	Inhibiting the proliferation of VSMCs by serving as an AP-1 inhibitor	(Hsieh et al., 2008a; Hsieh et al., 2008b)
HO-3867	10 µM	Human aortic SMCs	Inhibiting the proliferation of serum-stimulated VSMCs by inducing cell cycle arrest	Selvendiran et al. (2009)
Curcumin	1–50 μM	A10 cells	Inhibiting ET-1-induced mitogenic and proliferative signaling events in VSMCs	Kapakos et al. (2012)
Curcumin	5, 10, 20 μM	Primary rat VSMCs	Inhibiting Ang II-induced inflammation and proliferation of rat VSMCs	Li et al. (2017)
Nicotinate-curcumin	1 μM	VSMCs	Inhibiting Ang II-induced vascular smooth muscle cell phenotype switching	Sun et al. (2021)
Curcumin	10, 20, 40 μM	Primary mouse VSMCs	Inhibiting the proliferation and migration of vascular smooth muscle cells by targeting the chemerin/CMKLR1/LCN2 axis	He et al. (2021)
Curcumin	25 μM	Airway smooth muscle cells	Inhibiting the proliferation of cells by upregulating the expression of caveolin-1 and blocking the activation of the ERK pathway	Zeng et al. (2013)
Curcumin	10^ ^μM	A7R5 cells	Inhibiting the proliferation, migration and neointimal formation of VSMCs via activating miR-22	Zhang et al. (2020)
Curcumin	1, 5, 10, 20 μM	Primary rat VSMCs	Inducing growth inhibition in rat VSMCs by upregulation of HO-1	Pae et al. (2007)
Curcumin	Not Applicable	Primary pulmonary arterial smooth muscle cells	Promoting cell apoptosis; protecting mitochondrial function; suppressing the PI3K/AKT pathway	Chen et al. (2021)
Curcumin	20 μM	Mouse aortic smooth muscle cell line A7R5 cells	Inhibiting the phenotypic transformation, migration, and foaming of ox-LDL-treated VSMCs	Wang et al. (2021b)

## References

[B1] Acelajado M. C. Hughes Z. H. Oparil S. Calhoun D. A. (2019). Treatment of Resistant and Refractory Hypertension. Circ. Res. 124, 1061–1070. 10.1161/circresaha.118.312156 30920924 PMC6469348

[B2] Adahoun M. a. A. Al-Akhras M.-A. H. Jaafar M. S. Bououdina M. (2017). Enhanced Anti-cancer and Antimicrobial Activities of Curcumin Nanoparticles. Artif. Cell Nanomedicine, Biotechnol. 45, 98–107. 10.3109/21691401.2015.1129628 26747522

[B3] Aggarwal B. B. Kumar A. Bharti A. C. (2003). Anticancer Potential of Curcumin: Preclinical and Clinical Studies. Anticancer Res. 23, 363–398. 12680238

[B4] Ahangari N. Kargozar S. Ghayour‐Mobarhan M. Baino F. Pasdar A. Sahebkar A. (2019). Curcumin in Tissue Engineering: A Traditional Remedy for Modern Medicine. Biofactors 45, 135–151. 10.1002/biof.1474 30537039

[B5] Akbar M. U. Zia K. M. Nazir A. Iqbal J. Ejaz S. A. Akash M. S. H. (2018). Pluronic-Based Mixed Polymeric Micelles Enhance the Therapeutic Potential of Curcumin. AAPS PharmSciTech 19, 2719–2739. 10.1208/s12249-018-1098-9 29978290

[B6] Alappat L. Awad A. B. (2010). Curcumin and Obesity: Evidence and Mechanisms. Nutr. Rev. 68, 729–738. 10.1111/j.1753-4887.2010.00341.x 21091916

[B7] Alidadi M. Liberale L. Montecucco F. Majeed M. Al-Rasadi K. Banach M. (2021). Protective Effects of Curcumin on Endothelium: An Updated Review. Adv. Exp. Med. Biol. 1291, 103–119. 10.1007/978-3-030-56153-6_6 34331686

[B8] Allahverdian S. Chehroudi A. C. Mcmanus B. M. Abraham T. Francis G. A. (2014). Contribution of Intimal Smooth Muscle Cells to Cholesterol Accumulation and Macrophage-like Cells in Human Atherosclerosis. Circulation 129, 1551–1559. 10.1161/circulationaha.113.005015 24481950

[B9] Almenara C. C. P. Oliveira T. F. Padilha A. S. (2020). The Role of Antioxidants in the Prevention of Cadmium-Induced Endothelial Dysfunction. Cpd 26, 3667–3675. 10.2174/1381612826666200415172338 32294029

[B10] Alwi I. Santoso T. Suyono S. Sutrisna B. Suyatna F. D. Kresno S. B. (2008). The Effect of Curcumin on Lipid Level in Patients with Acute Coronary Syndrome. Acta Med. Indones 40, 201–210. 19151449

[B11] Amin F. Gilani A.-H. Mehmood M. H. Siddiqui B. S. Khatoon N. (2015). Coadministration of Black Seeds and Turmeric Shows Enhanced Efficacy in Preventing Metabolic Syndrome in Fructose-Fed Rats. J. Cardiovasc. Pharmacol. 65, 176–183. 10.1097/fjc.0000000000000179 25384193

[B12] Anand P. Kunnumakkara A. B. Newman R. A. Aggarwal B. B. (2007). Bioavailability of Curcumin: Problems and Promises. Mol. Pharmaceutics 4, 807–818. 10.1021/mp700113r 17999464

[B13] Anderson W. P. Kett M. M. Stevenson K. M. Edgley A. J. Denton K. M. Fitzgerald S. M. (2000). Renovascular Hypertension. Hypertension 36, 648–652. 10.1161/01.hyp.36.4.648 11040252

[B14] Arnett D. K. Blumenthal R. S. Albert M. A. Buroker A. B. Goldberger Z. D. Hahn E. J. (20192019). 2019 ACC/AHA Guideline on the Primary Prevention of Cardiovascular Disease: A Report of the American College of Cardiology/American Heart Association Task Force on Clinical Practice Guidelines. J. Am. Coll. Cardiol. 74, e177–e646. 10.1016/j.jacc.2019.03.010 PMC7685565 30894318

[B15] Artiach G. Carracedo M. Clària J. Laguna-Fernandez A. Bäck M. (2018). Opposing Effects on Vascular Smooth Muscle Cell Proliferation and Macrophage-Induced Inflammation Reveal a Protective Role for the Proresolving Lipid Mediator Receptor ChemR23 in Intimal Hyperplasia. Front. Pharmacol. 9, 1327. 10.3389/fphar.2018.01327 30515096 PMC6255922

[B16] Aryal S. R. Siddiqui M. Sharifov O. F. Coffin M. D. Zhang B. Gaddam K. K. (2021). Spironolactone Reduces Aortic Stiffness in Patients with Resistant Hypertension Independent of Blood Pressure Change. J. Am. Heart Assoc. 10, e019434. 10.1161/JAHA.120.019434 34459249 PMC8649301

[B17] Bahramsoltani R. Rahimi R. Farzaei M. H. (2017). Pharmacokinetic Interactions of Curcuminoids with Conventional Drugs: A Review. J. Ethnopharmacology 209, 1–12. 10.1016/j.jep.2017.07.022 28734960

[B18] Bakris G. Yang Y. F. Pitt B. (2020). Mineralocorticoid Receptor Antagonists for Hypertension Management in Advanced Chronic Kidney Disease. Hypertension 76, 144–149. 10.1161/hypertensionaha.120.15199 32520623

[B19] Barber-Chamoux N. Milenkovic D. Verny M. A. Habauzit V. Pereira B. Lambert C. (2018). Substantial Variability across Individuals in the Vascular and Nutrigenomic Response to an Acute Intake of Curcumin: A Randomized Controlled Trial. Mol. Nutr. Food Res. 62. 10.1002/mnfr.201700418 29034576

[B20] Barton M. Yanagisawa M. (2019). Endothelin: 30 Years from Discovery to Therapy. Hypertension 74, 1232–1265. 10.1161/hypertensionaha.119.12105 31679425

[B21] Blacher J. Guerin A. P. Pannier B. Marchais S. J. Safar M. E. London G. M. (1999). Impact of Aortic Stiffness on Survival in End-Stage Renal Disease. Circulation 99, 2434–2439. 10.1161/01.cir.99.18.2434 10318666

[B22] Boari G. E. Rizzoni D. De Ciuceis C. Porteri E. Avanzi D. Platto C. (2010). Structural Alterations in Subcutaneous Small Resistance Arteries Predict Changes in the Renal Function of Hypertensive Patients. J. Hypertens. 28, 1951–1958. 10.1097/hjh.0b013e32833c2177 20577125

[B23] Bomzon A. Holt S. Moore K. (1997). Bile Acids, Oxidative Stress, and Renal Function in Biliary Obstruction. Semin. Nephrol. 17, 549–562. 9353865

[B24] Boonla O. Kukongviriyapan U. Pakdeechote P. Kukongviriyapan V. Pannangpetch P. Prachaney P. (2014). Curcumin Improves Endothelial Dysfunction and Vascular Remodeling in 2K-1C Hypertensive Rats by Raising Nitric Oxide Availability and Reducing Oxidative Stress. Nitric Oxide 42, 44–53. 10.1016/j.niox.2014.09.001 25194767

[B25] Botts S. R. Fish J. E. Howe K. L. (2021). Dysfunctional Vascular Endothelium as a Driver of Atherosclerosis: Emerging Insights into Pathogenesis and Treatment. Front. Pharmacol. 12, 787541. 10.3389/fphar.2021.787541 35002720 PMC8727904

[B26] Briet M. Schiffrin E. L. (2013). Treatment of Arterial Remodeling in Essential Hypertension. Curr. Hypertens. Rep. 15, 3–9. 10.1007/s11906-012-0325-0 23250721

[B27] Bronte E. Coppola G. Di Miceli R. Sucato V. Russo A. Novo S. (2013). Role of Curcumin in Idiopathic Pulmonary Arterial Hypertension Treatment: a New Therapeutic Possibility. Med. Hypotheses 81, 923–926. 10.1016/j.mehy.2013.08.016 24054817

[B28] Brown I. A. M. Diederich L. Good M. E. Delalio L. J. Murphy S. A. Cortese-Krott M. M. (2018). Vascular Smooth Muscle Remodeling in Conductive and Resistance Arteries in Hypertension. Atvb 38, 1969–1985. 10.1161/atvbaha.118.311229 PMC6205219 30354262

[B29] Campbell M. S. Ouyang A. I.M. K. Charnigo R. J. Westgate P. M. Fleenor B. S. (2019). Influence of Enhanced Bioavailable Curcumin on Obesity-Associated Cardiovascular Disease Risk Factors and Arterial Function: A Double-Blinded, Randomized, Controlled Trial. Nutrition 62, 135–139. 10.1016/j.nut.2019.01.002 30889454

[B30] Canale M. P. Noce A. Di Lauro M. Marrone G. Cantelmo M. Cardillo C. (2021). Gut Dysbiosis and Western Diet in the Pathogenesis of Essential Arterial Hypertension: A Narrative Review. Nutrients 13, 1162. 10.3390/nu13041162 33915885 PMC8066853

[B31] Carey R. M. Muntner P. Bosworth H. B. Whelton P. K. (2018). Prevention and Control of Hypertension. J. Am. Coll. Cardiol. 72, 1278–1293. 10.1016/j.jacc.2018.07.008 30190007 PMC6481176

[B32] Chazova I. Loyd J. E. Zhdanov V. S. Newman J. H. Belenkov Y. Meyrick B. (1995). Pulmonary Artery Adventitial Changes and Venous Involvement in Primary Pulmonary Hypertension. Am. J. Pathol. 146, 389–397. 7856750 PMC1869854

[B33] Chen H.-W. Huang H.-C. (1998). Effect of Curcumin on Cell Cycle Progression and Apoptosis in Vascular Smooth Muscle Cells. Br. J. Pharmacol. 124, 1029–1040. 10.1038/sj.bjp.0701914 9720770 PMC1565483

[B34] Chen J. Jiang W. Zhu F. Wang Q. Yang H. Wu J. (2021). Curcumin Improves Pulmonary Hypertension Rats by Regulating Mitochondrial Function. Biomed. Res. Int. 2021, 1078019. 10.1155/2021/1078019 34497845 PMC8421153

[B35] Chen X. Chen X. Shi X. Gao Z. Guo Z. (2020). Curcumin Attenuates Endothelial Cell Fibrosis through Inhibiting Endothelial-Interstitial Transformation. Clin. Exp. Pharmacol. Physiol. 47, 1182–1192. 10.1111/1440-1681.13271 32020664 PMC7318201

[B36] Chuengsamarn S. Rattanamongkolgul S. Luechapudiporn R. Phisalaphong C. Jirawatnotai S. (2012). Curcumin Extract for Prevention of Type 2 Diabetes. Diabetes Care 35, 2121–2127. 10.2337/dc12-0116 22773702 PMC3476912

[B37] Cox F. F. Misiou A. Vierkant A. Ale-Agha N. Grandoch M. Haendeler J. (2022). Protective Effects of Curcumin in Cardiovascular Diseases-Impact on Oxidative Stress and Mitochondria. Cells 11, 342. 10.3390/cells11030342 35159155 PMC8833931

[B38] Cox R. H. Rusch N. J. (2002). New Expression Profiles of Voltage-Gated Ion Channels in Arteries Exposed to High Blood Pressure. Microcirculation 9, 243–257. 10.1080/mic.9.4.243.257 12152102

[B39] Cui J. Melista E. Chazaro I. Zhang Y. Zhou X. Manolis A. J. (2005). Sequence Variation of Bradykinin Receptors B1 and B2 and Association with Hypertension. J. Hypertens. 23, 55–62. 10.1097/00004872-200501000-00013 15643125

[B40] Czyzynska-Cichon I. Janik-Hazuka M. Szafraniec-Szczęsny J. Jasinski K. Węglarz W. P. Zapotoczny S. (2021). Low Dose Curcumin Administered in Hyaluronic Acid-Based Nanocapsules Induces Hypotensive Effect in Hypertensive Rats. Ijn 16, 1377–1390. 10.2147/ijn.s291945 33658778 PMC7917338

[B41] Deciuceis C. Porteri E. Rizzoni D. Rizzardi N. Paiardi S. Boari G. (2007). Structural Alterations of Subcutaneous Small-Resistance Arteries May Predict Major Cardiovascular Events in Patients with Hypertension. Am. J. Hypertens. 20, 846–852. 10.1016/j.amjhyper.2007.03.016 17679031

[B42] Devadasu V. R. Wadsworth R. M. Ravi Kumar M. N. V. (2012). Tissue Localization of Nanoparticles Is Altered Due to Hypoxia Resulting in Poor Efficacy of Curcumin Nanoparticles in Pulmonary Hypertension. Eur. J. Pharmaceutics Biopharmaceutics 80, 578–584. 10.1016/j.ejpb.2011.12.008 22227367

[B43] Dhaun N. Webb D. J. (2019). Endothelins in Cardiovascular Biology and Therapeutics. Nat. Rev. Cardiol. 16, 491–502. 10.1038/s41569-019-0176-3 30867577

[B44] Di L. Maiseyeu A. (2021). Low-density Lipoprotein Nanomedicines: Mechanisms of Targeting, Biology, and Theranostic Potential. Drug Deliv. 28, 408–421. 10.1080/10717544.2021.1886199 33594923 PMC7894439

[B45] Du Preez R. Pahl J. Arora M. Ravi Kumar M. N. V. Brown L. Panchal S. K. (2019). Low-Dose Curcumin Nanoparticles Normalise Blood Pressure in Male Wistar Rats with Diet-Induced Metabolic Syndrome. Nutrients 11, 1542. 10.3390/nu11071542 PMC6682951 31288419

[B46] El-Bassossy H. M. Hassan N. Zakaria M. N. M. (2014). Heme Oxygenase-1 Alleviates Vascular Complications Associated with Metabolic Syndrome: Effect on Endothelial Dependent Relaxation and NO Production. Chemico-Biological Interactions 223, 109–115. 10.1016/j.cbi.2014.09.014 25268984

[B47] Fadus M. C. Lau C. Bikhchandani J. Lynch H. T. (2017). Curcumin: An Age-Old Anti-inflammatory and Anti-neoplastic Agent. J. Traditional Complement. Med. 7, 339–346. 10.1016/j.jtcme.2016.08.002 PMC5506636 28725630

[B48] Fan J. Li X. Yan Y.-W. Tian X.-H. Hou W.-J. Tong H. (2012). Curcumin Attenuates Rat Thoracic Aortic Aneurysm Formation by Inhibition of the C-Jun N-Terminal Kinase Pathway and Apoptosis. Nutrition 28, 1068–1074. 10.1016/j.nut.2012.02.006 22840386

[B49] Farhangkhoee H. Khan Z. A. Mukherjee S. Cukiernik M. Barbin Y. P. Karmazyn M. (2003). Heme Oxygenase in Diabetes-Induced Oxidative Stress in the Heart. J. Mol. Cell Cardiol. 35, 1439–1448. 10.1016/j.yjmcc.2003.09.007 14654370

[B50] Foley A. Steinberg B. E. Goldenberg N. M. (2021). Inflammasome Activation in Pulmonary Arterial Hypertension. Front. Med. (Lausanne) 8, 826557. 10.3389/fmed.2021.826557 35096915 PMC8792742

[B51] Forouzanfar M. H. Liu P. Roth G. A. Ng M. Biryukov S. Marczak L. (2017). Global Burden of Hypertension and Systolic Blood Pressure of at Least 110 to 115 Mm Hg, 1990-2015. Jama 317, 165–182. 10.1001/jama.2016.19043 28097354

[B52] Friese R. S. Altshuler A. E. Zhang K. Miramontes-Gonzalez J. P. Hightower C. M. Jirout M. L. (2013). MicroRNA-22 and Promoter Motif Polymorphisms at the Chga Locus in Genetic Hypertension: Functional and Therapeutic Implications for Gene Expression and the Pathogenesis of Hypertension. Hum. Mol. Genet. 22, 3624–3640. 10.1093/hmg/ddt213 23674521 PMC3749858

[B53] Fu Y.-S. Chen T.-H. Weng L. Huang L. Lai D. Weng C.-F. (2021). Pharmacological Properties and Underlying Mechanisms of Curcumin and Prospects in Medicinal Potential. Biomed. Pharmacother. 141, 111888. 10.1016/j.biopha.2021.111888 34237598

[B54] Garg A. X. Devereaux P. J. Hill A. Sood M. Aggarwal B. Dubois L. (2018). Oral Curcumin in Elective Abdominal Aortic Aneurysm Repair: a Multicentre Randomized Controlled Trial. Cmaj 190, E1273–e1280. 10.1503/cmaj.180510 30373740 PMC6205831

[B55] Girchev R. Backer A. Markova P. Kramer H. J. (2006). Renal Endothelin System and Excretory Function in Wistar-Kyoto and Long-Evans Rats. Acta Physiol. 186, 67–76. 10.1111/j.1748-1716.2005.01501.x 16497181

[B56] Gong L. Lei Y. Liu Y. Tan F. Li S. Wang X. (2019). Vaccarin Prevents Ox-LDL-Induced HUVEC EndMT, Inflammation and Apoptosis by Suppressing ROS/p38 MAPK Signaling. Am. J. Transl Res. 11, 2140–2154. 31105824 PMC6511755

[B57] Gouda A. S. Adbelruhman F. G. Elbendary R. N. Alharbi F. A. Alhamrani S. Q. Mégarbane B. (2021). A Comprehensive Insight into the Role of Zinc Deficiency in the Renin-Angiotensin and Kinin-Kallikrein System Dysfunctions in COVID-19 Patients. Saudi J. Biol. Sci. 28, 3540–3547. 10.1016/j.sjbs.2021.03.027 33746538 PMC7962980

[B58] Goyal A. Petersen J. L. Mahaffey K. W. (2004). The Evaluation and Management of Dyslipidemia and Impaired Glucose Metabolism during Acute Coronary Syndromes. Curr. Cardiol. Rep. 6, 300–307. 10.1007/s11886-004-0080-1 15182608

[B59] Gumprecht J. Domek M. Lip G. Y. H. Shantsila A. (2019). Invited Review: Hypertension and Atrial Fibrillation: Epidemiology, Pathophysiology, and Implications for Management. J. Hum. Hypertens. 33, 824–836. 10.1038/s41371-019-0279-7 31690818

[B60] Gupta S. C. Patchva S. Aggarwal B. B. (2013). Therapeutic Roles of Curcumin: Lessons Learned from Clinical Trials. Aaps j 15, 195–218. 10.1208/s12248-012-9432-8 23143785 PMC3535097

[B61] Gurha P. Abreu-Goodger C. Wang T. Ramirez M. O. Drumond A. L. Van Dongen S. (2012). Targeted Deletion of microRNA-22 Promotes Stress-Induced Cardiac Dilation and Contractile Dysfunction. Circulation 125, 2751–2761. 10.1161/circulationaha.111.044354 22570371 PMC3503489

[B62] Hadi A. Pourmasoumi M. Ghaedi E. Sahebkar A. (2019). The Effect of Curcumin/Turmeric on Blood Pressure Modulation: A Systematic Review and Meta-Analysis. Pharmacol. Res. 150, 104505. 10.1016/j.phrs.2019.104505 31647981

[B63] Han Y. Sun H.-J. Tong Y. Chen Y.-Z. Ye C. Qiu Y. (2019). Curcumin Attenuates Migration of Vascular Smooth Muscle Cells via Inhibiting NFκB-Mediated NLRP3 Expression in Spontaneously Hypertensive Rats. J. Nutr. Biochem. 72, 108212. 10.1016/j.jnutbio.2019.07.003 31473513

[B64] Harrison D. G. Coffman T. M. Wilcox C. S. (2021). Pathophysiology of Hypertension. Circ. Res. 128, 847–863. 10.1161/circresaha.121.318082 33793328 PMC8023760

[B65] Hassan N. El-Bassossy H. M. Zakaria M. N. M. (2013). Heme Oxygenase-1 Induction Protects against Hypertension Associated with Diabetes: Effect on Exaggerated Vascular Contractility. Naunyn-schmiedeberg's Arch. Pharmacol. 386, 217–226. 10.1007/s00210-012-0822-3 23254361

[B66] He Y. Wang R. Zhang P. Yan J. Gong N. Li Y. (2021). Curcumin Inhibits the Proliferation and Migration of Vascular Smooth Muscle Cells by Targeting the Chemerin/CMKLR1/LCN2 axis. Aging 13, 13859–13875. 10.18632/aging.202980 34029211 PMC8202847

[B67] Heldin C.-H. Westermark B. (1999). Mechanism of Action and *In Vivo* Role of Platelet-Derived Growth Factor. Physiol. Rev. 79, 1283–1316. 10.1152/physrev.1999.79.4.1283 10508235

[B68] Hesari M. Mohammadi P. Khademi F. Shackebaei D. Momtaz S. Moasefi N. (2021). Current Advances in the Use of Nanophytomedicine Therapies for Human Cardiovascular Diseases. Ijn 16, 3293–3315. 10.2147/ijn.s295508 34007178 PMC8123960

[B69] Hlavačková L. Janegová A. Uličná O. Janega P. Cerná A. Babál P. (2011). Spice up the Hypertension Diet - Curcumin and Piperine Prevent Remodeling of Aorta in Experimental L-NAME Induced Hypertension. Nutr. Metab. (Lond) 8, 72. 22005253 10.1186/1743-7075-8-72 PMC3214182

[B70] Hodaei H. Adibian M. Nikpayam O. Hedayati M. Sohrab G. (2019). The Effect of Curcumin Supplementation on Anthropometric Indices, Insulin Resistance and Oxidative Stress in Patients with Type 2 Diabetes: a Randomized, Double-Blind Clinical Trial. Diabetol. Metab. Syndr. 11, 41. 10.1186/s13098-019-0437-7 31149032 PMC6537430

[B71] Hosseini A. Penson P. E. Cicero A. F. G. Golledge J. Al-Rasadi K. Jamialahmadi T. (2021). Potential Benefits of Phytochemicals for Abdominal Aortic Aneurysm. Cmc 28, 8595–8607. 10.2174/0929867328666210614113116 34126879

[B72] Hsieh H.-L. Sun C.-C. Wang T.-S. Yang C.-M. (2008a). PKC-δ/c-Src-mediated EGF Receptor Transactivation Regulates Thrombin-Induced COX-2 Expression and PGE2 Production in Rat Vascular Smooth Muscle Cells. Biochim. Biophys. Acta (Bba) - Mol. Cel Res. 1783, 1563–1575. 10.1016/j.bbamcr.2008.03.016 18452714

[B73] Hsieh H.-L. Sun C.-C. Wu C.-B. Wu C.-Y. Tung W.-H. Wang H.-H. (2008b). Sphingosine 1-phosphate Induces EGFR Expression via Akt/NF-Κb and ERK/AP-1 Pathways in Rat Vascular Smooth Muscle Cells. J. Cel. Biochem. 103, 1732–1746. 10.1002/jcb.21563 17902169

[B74] Hu X. Yang F.-F. Wei X.-L. Yao G.-Y. Liu C.-Y. Zheng Y. (2017). Curcumin Acetate Nanocrystals for Sustained Pulmonary Delivery: Preparation, Characterization and *In Vivo* Evaluation. J. Biomed. Nanotechnol 13, 99–109. 10.1166/jbn.2017.2326 29373003

[B75] Hua Y. Dolence J. Ramanan S. Ren J. Nair S. (2013). Bisdemethoxycurcumin Inhibits PDGF-Induced Vascular Smooth Muscle Cell Motility and Proliferation. Mol. Nutr. Food Res. 57, 1611–1618. 10.1002/mnfr.201200852 23554078 PMC3844678

[B76] Huang H. C. Jan T. R. Yeh S. F. (1992). Inhibitory Effect of Curcumin, an Anti-inflammatory Agent, on Vascular Smooth Muscle Cell Proliferation. Eur. J. Pharmacol. 221, 381–384. 10.1016/0014-2999(92)90727-l 1426014

[B77] Huang Z.-P. Chen J. Seok H. Y. Zhang Z. Kataoka M. Hu X. (2013). MicroRNA-22 Regulates Cardiac Hypertrophy and Remodeling in Response to Stress. Circ. Res. 112, 1234–1243. 10.1161/circresaha.112.300682 23524588 PMC3720677

[B78] Humbert M. Montani D. Perros F. Dorfmüller P. Adnot S. Eddahibi S. (2008). Endothelial Cell Dysfunction and Cross Talk between Endothelium and Smooth Muscle Cells in Pulmonary Arterial Hypertension. Vasc. Pharmacol. 49, 113–118. 10.1016/j.vph.2008.06.003 18606248

[B79] Huo M. Cao X. Zhang H. Lau C. W. Hong H. Chen F. M. (2021). Loss of Myeloid Bmal1 Exacerbates Hypertensive Vascular Remodelling through Interaction with STAT6 in Mice. Cardiovasc. Res. 10.1093/cvr/cvab336 34726702

[B80] Hussain Z. Thu H. E. Ng S.-F. Khan S. Katas H. (2017). Nanoencapsulation, an Efficient and Promising Approach to Maximize Wound Healing Efficacy of Curcumin: A Review of New Trends and State-Of-The-Art. Colloids Surf. B: Biointerfaces 150, 223–241. 10.1016/j.colsurfb.2016.11.036 27918967

[B81] James P. A. Oparil S. Carter B. L. Cushman W. C. Dennison-Himmelfarb C. Handler J. (2014). 2014 Evidence-Based Guideline for the Management of High Blood Pressure in Adults. Jama 311, 507–520. 10.1001/jama.2013.284427 24352797

[B82] Jeffery T. K. Morrell N. W. (2002). Molecular and Cellular Basis of Pulmonary Vascular Remodeling in Pulmonary Hypertension. Prog. Cardiovasc. Dis. 45, 173–202. 10.1053/pcad.2002.130041 12525995

[B83] Jouen-Tachoire T. R. H. Tucker S. J. Tammaro P. (2021). Ion Channels as Convergence Points in the Pathology of Pulmonary Arterial Hypertension. Biochem. Soc. Trans. 49, 1855–1865. 10.1042/bst20210538 34346486 PMC8421048

[B84] Kang G. Kong P.-J. Yuh Y.-J. Lim S.-Y. Yim S.-V. Chun W. (2004). Curcumin Suppresses Lipopolysaccharide-Induced Cyclooxygenase-2 Expression by Inhibiting Activator Protein 1 and Nuclear Factor κB Bindings in BV2 Microglial Cells. J. Pharmacol. Sci. 94, 325–328. 10.1254/jphs.94.325 15037818

[B85] Kapakos G. Youreva V. Srivastava A. K. (2012). Attenuation of Endothelin-1-Induced PKB and ERK1/2 Signaling, as Well as Egr-1 Expression, by Curcumin in A-10 Vascular Smooth Muscle Cells. Can. J. Physiol. Pharmacol. 90, 1277–1285. 10.1139/y2012-059 22913328

[B86] Karaki H. Ozaki H. Hori M. Mitsui-Saito M. Amano K. Harada K. (1997). Calcium Movements, Distribution, and Functions in Smooth Muscle. Pharmacol. Rev. 49, 157–230. 9228665

[B87] Karaki H. Weiss G. B. (1988). Calcium Release in Smooth Muscle. Life Sci. 42, 111–122. 10.1016/0024-3205(88)90674-1 2447464

[B88] Kedzierski R. M. Grayburn P. A. Kisanuki Y. Y. Williams C. S. Hammer R. E. Richardson J. A. (2003). Cardiomyocyte-specific Endothelin A Receptor Knockout Mice Have normal Cardiac Function and an Unaltered Hypertrophic Response to Angiotensin II and Isoproterenol. Mol. Cel Biol 23, 8226–8232. 10.1128/mcb.23.22.8226-8232.2003 PMC262340 14585980

[B89] Kennedy A. J. Yang P. Read C. Kuc R. E. Yang L. Taylor E. J. (2016). Chemerin Elicits Potent Constrictor Actions via Chemokine-like Receptor 1 (CMKLR1), Not G-Protein-Coupled Receptor 1 (GPR1), in Human and Rat Vasculature. J. Am. Heart Assoc. 5. 10.1161/JAHA.116.004421 PMC5121526 27742615

[B90] Khajehdehi P. Pakfetrat M. Javidnia K. Azad F. Malekmakan L. Nasab M. H. (2011). Oral Supplementation of Turmeric Attenuates Proteinuria, Transforming Growth Factor-β and Interleukin-8 Levels in Patients with Overt Type 2 Diabetic Nephropathy: A Randomized, Double-Blind and Placebo-Controlled Study. Scand. J. Urol. Nephrol. 45, 365–370. 10.3109/00365599.2011.585622 21627399

[B91] Khajehdehi P. Zanjaninejad B. Aflaki E. Nazarinia M. Azad F. Malekmakan L. (2012). Oral Supplementation of Turmeric Decreases Proteinuria, Hematuria, and Systolic Blood Pressure in Patients Suffering from Relapsing or Refractory Lupus Nephritis: a Randomized and Placebo-Controlled Study. J. Ren. Nutr. 22, 50–57. 10.1053/j.jrn.2011.03.002 21742514

[B92] Khayyal M. T. El-Hazek R. M. El-Sabbagh W. A. Frank J. Behnam D. Abdel-Tawab M. (2018). Micellar Solubilisation Enhances the Antiinflammatory Activities of Curcumin and Boswellic Acids in Rats with Adjuvant-Induced Arthritis. Nutrition 54, 189–196. 10.1016/j.nut.2018.03.055 30048884

[B93] Kim H. W. Belin de Chantemèle E. J. Weintraub N. L. (2019). Perivascular Adipocytes in Vascular Disease. Atvb 39, 2220–2227. 10.1161/atvbaha.119.312304 PMC6812617 31510794

[B94] Kim J.-Y. Cho H.-J. Sir J.-J. Kim B.-K. Hur J. Youn S.-W. (2009). Sulfasalazine Induces Haem Oxygenase-1 via ROS-dependent Nrf2 Signalling, Leading to Control of Neointimal Hyperplasia. Cardiovasc. Res. 82, 550–560. 10.1093/cvr/cvp072 19234301

[B95] Kintsurashvili E. Duka I. Gavras I. Johns C. Farmakiotis D. Gavras H. (2001). Effects of ANG II on Bradykinin Receptor Gene Expression in Cardiomyocytes and Vascular Smooth Muscle Cells. Am. J. Physiology-Heart Circulatory Physiol. 281, H1778–H1783. 10.1152/ajpheart.2001.281.4.h1778 11557571

[B96] Kostov K. (2021). The Causal Relationship between Endothelin-1 and Hypertension: Focusing on Endothelial Dysfunction, Arterial Stiffness, Vascular Remodeling, and Blood Pressure Regulation. Life (Basel) 11. 10.3390/life11090986 PMC8472034 34575135

[B97] Kruangtip O. Chootip K. Temkitthawon P. Changwichit K. Chuprajob T. Changtam C. (2015). Curcumin Analogues Inhibit Phosphodiesterase-5 and Dilate Rat Pulmonary Arteries. J. Pharm. Pharmacol. 67, 87–95. 10.1111/jphp.12302 25176340

[B98] Kukongviriyapan U. Apaijit K. Kukongviriyapan V. (2016). Oxidative Stress and Cardiovascular Dysfunction Associated with Cadmium Exposure: Beneficial Effects of Curcumin and Tetrahydrocurcumin. Tohoku J. Exp. Med. 239, 25–38. 10.1620/tjem.239.25 27151191

[B99] Kukongviriyapan U. Pannangpetch P. Kukongviriyapan V. Donpunha W. Sompamit K. Surawattanawan P. (2014). Curcumin Protects against Cadmium-Induced Vascular Dysfunction, Hypertension and Tissue Cadmium Accumulation in Mice. Nutrients 6, 1194–1208. 10.3390/nu6031194 24662163 PMC3967187

[B100] Kunati S. R. Yang S. William B. M. Xu Y. (2018). An LC-MS/MS Method for Simultaneous Determination of Curcumin, Curcumin Glucuronide and Curcumin Sulfate in a Phase II Clinical Trial. J. Pharm. Biomed. Anal. 156, 189–198. 10.1016/j.jpba.2018.04.034 29727780

[B101] Lao C. D. Ruffin M. T. Normolle D. Heath D. D. Murray S. I. Bailey J. M. (2006). Dose Escalation of a Curcuminoid Formulation. BMC Complement. Altern. Med. 6, 10. 10.1186/1472-6882-6-10 16545122 PMC1434783

[B102] Laurent S. Boutouyrie P. (2015). The Structural Factor of Hypertension. Circ. Res. 116, 1007–1021. 10.1161/circresaha.116.303596 25767286

[B103] Lee K.-H. Abas F. Alitheen N. B. M. Shaari K. Lajis N. H. Ahmad S. (2011). A Curcumin Derivative, 2,6-Bis(2,5-Dimethoxybenzylidene)-Cyclohexanone (BDMC33) Attenuates Prostaglandin E2 Synthesis via Selective Suppression of Cyclooxygenase-2 in IFN-g/LPS-Stimulated Macrophages. Molecules 16, 9728–9738. 10.3390/molecules16119728 22113581 PMC6264440

[B104] Lee K.-H. Chow Y.-L. Sharmili V. Abas F. Alitheen N. B. M. Shaari K. (2012). BDMC33, A Curcumin Derivative Suppresses Inflammatory Responses in Macrophage-like Cellular System: Role of Inhibition in NF-Κb and MAPK Signaling Pathways. Ijms 13, 2985–3008. 10.3390/ijms13032985 22489138 PMC3317699

[B105] Lee T.-S. Chang C.-C. Zhu Y. Shyy J. Y.-J. (2004). Simvastatin Induces Heme Oxygenase-1. Circulation 110, 1296–1302. 10.1161/01.cir.0000140694.67251.9c 15337692

[B106] Li H.-B. Xu M.-L. Du M.-M. Yu X.-J. Bai J. Xia W.-J. (2021). Curcumin Ameliorates Hypertension via Gut-Brain Communication in Spontaneously Hypertensive Rat. Toxicol. Appl. Pharmacol. 429, 115701. 10.1016/j.taap.2021.115701 34453990

[B107] Li H.-B. Yang T. Richards E. M. Pepine C. J. Raizada M. K. (2020). Maternal Treatment with Captopril Persistently Alters Gut-Brain Communication and Attenuates Hypertension of Male Offspring. Hypertension 75, 1315–1324. 10.1161/hypertensionaha.120.14736 32200676 PMC7145738

[B108] Li H.-Y. Yang M. Li Z. Meng Z. (2017). Curcumin Inhibits Angiotensin II-Induced Inflammation and Proliferation of Rat Vascular Smooth Muscle Cells by Elevating PPAR-γ Activity and Reducing Oxidative Stress. Int. J. Mol. Med. 39, 1307–1316. 10.3892/ijmm.2017.2924 28339005

[B109] Li J. L. Fan Y. Y. Ye G. H. Dong M. W. Lin K. Z. Li F. (2014). Study on the Mechanism of How Curcumin Improves Pulmonary Vascular Remodeling Associated with Chronic Pulmonary Arterial Hypertension. Zhongguo Ying Yong Sheng Li Xue Za Zhi 30, 451–455. 25571640

[B110] Li Y. Tian D. Zhu C. Ren L. (2016). Demethoxycurcumin Preserves Renovascular Function by Downregulating COX-2 Expression in Hypertension. Oxid Med. Cel Longev 2016, 9045736. 10.1155/2016/9045736 PMC5220467 28105253

[B111] Li Y. Lui K. O. Zhou B. (2018). Reassessing Endothelial-To-Mesenchymal Transition in Cardiovascular Diseases. Nat. Rev. Cardiol. 15, 445–456. 10.1038/s41569-018-0023-y 29748594

[B112] Lin Q. Wang L. X. Chen S. X. Zhou X. F. Huang X. Y. Fan X. F. (2006). Effect of Curcumin on Pulmonary Hypertension and wall Collagen of Pulmonary Arterioles of Chronic Hypoxic Hypercapnic Rats. Zhongguo Ying Yong Sheng Li Xue Za Zhi 22, 257–261. 21158062

[B113] Ling L. Chen D. Tong Y. Zang Y.-H. Ren X.-S. Zhou H. (2018). Fibronectin Type III Domain Containing 5 Attenuates NLRP3 Inflammasome Activation and Phenotypic Transformation of Adventitial Fibroblasts in Spontaneously Hypertensive Rats. J. Hypertens. 36, 1104–1114. 10.1097/hjh.0000000000001654 29303830

[B114] Liu H. Xiong W. Luo Y. Chen H. He Y. Cao Y. (2019). Adipokine Chemerin Stimulates Progression of Atherosclerosis in ApoE-/- Mice. Biomed. Res. Int. 2019, 7157865. 10.1155/2019/7157865 31781638 PMC6875193

[B115] Liu Y. Dolence J. Ren J. Rao M. Sreejayan N. (2008). Inhibitory Effect of Dehydrozingerone on Vascular Smooth Muscle Cell Function. J. Cardiovasc. Pharmacol. 52, 422–429. 10.1097/fjc.0b013e31818aed93 19033821

[B116] Lu H. Du W. Ren L. Hamblin M. H. Becker R. C. Chen Y. E. (2021). Vascular Smooth Muscle Cells in Aortic Aneurysm: From Genetics to Mechanisms. J. Am. Heart Assoc. 10, e023601. 10.1161/JAHA.121.023601 34796717 PMC9075263

[B117] Lu Q.-B. Wan M.-Y. Wang P.-Y. Zhang C.-X. Xu D.-Y. Liao X. (2018a). Chicoric Acid Prevents PDGF-BB-Induced VSMC Dedifferentiation, Proliferation and Migration by Suppressing ROS/NFκB/mTOR/P70S6K Signaling cascade. Redox Biol. 14, 656–668. 10.1016/j.redox.2017.11.012 29175753 PMC5716955

[B118] Lu Q.-B. Wang H.-P. Tang Z.-H. Cheng H. Du Q. Wang Y.-B. (2018b). Nesfatin-1 Functions as a Switch for Phenotype Transformation and Proliferation of VSMCs in Hypertensive Vascular Remodeling. Biochim. Biophys. Acta (Bba) - Mol. Basis Dis. 1864, 2154–2168. 10.1016/j.bbadis.2018.04.002 29627363

[B119] Lu X. Gong J. Dennery P. A. Yao H. (2019). Endothelial-to-mesenchymal Transition: Pathogenesis and Therapeutic Targets for Chronic Pulmonary and Vascular Diseases. Biochem. Pharmacol. 168, 100–107. 10.1016/j.bcp.2019.06.021 31251941 PMC6733623

[B120] Luo P. Qiu B. (2022). The Role of Immune Cells in Pulmonary Hypertension: Focusing on Macrophages. Hum. Immunol. 83, 153–163. 10.1016/j.humimm.2021.11.006 34844784

[B121] Lüscher T. F. Barton M. (2000). Endothelins and Endothelin Receptor Antagonists: Therapeutic Considerations for a Novel Class of Cardiovascular Drugs. Circulation 102, 2434–2440. 11067800 10.1161/01.cir.102.19.2434

[B122] Lynch I. J. Welch A. K. Gumz M. L. Kohan D. E. Cain B. D. Wingo C. S. (2015). Effect of Mineralocorticoid Treatment in Mice with Collecting Duct-specific Knockout of Endothelin-1. Am. J. Physiology-Renal Physiol. 309, F1026–F1034. 10.1152/ajprenal.00220.2015 PMC4683304 26400543

[B123] Mahmoud M. F. El Bassossy H. M. (2014). Curcumin Attenuates Fructose-Induced Vascular Dysfunction of Isolated Rat Thoracic Aorta Rings. Pharm. Biol. 52, 972–977. 10.3109/13880209.2013.874465 24611676

[B124] Majithiya J. B. Balaraman R. (2005). Time-dependent Changes in Antioxidant Enzymes and Vascular Reactivity of Aorta in Streptozotocin-Induced Diabetic Rats Treated with Curcumin. J. Cardiovasc. Pharmacol. 46, 697–705. 10.1097/01.fjc.0000183720.85014.24 16220078

[B125] Maradana M. R. Thomas R. O'sullivan B. J. (2013). Targeted Delivery of Curcumin for Treating Type 2 Diabetes. Mol. Nutr. Food Res. 57, 1550–1556. 10.1002/mnfr.201200791 23495213

[B126] Marceau F. Bachelard H. Charest-Morin X. Hébert J. Rivard G. E. (2020). *In Vitro* Modeling of Bradykinin-Mediated Angioedema States. Pharmaceuticals 13, 201. 10.3390/ph13090201 PMC7559923 32824891

[B127] Mccoy E. K. Lisenby K. M. (2021). Aprocitentan (A Dual Endothelin-Receptor Antagonist) for Treatment-Resistant Hypertension. J. Cardiovasc. Pharmacol. 77, 699–706. 10.1097/fjc.0000000000001023 34001723

[B128] Meydani M. Hasan S. T. (2010). Dietary Polyphenols and Obesity. Nutrients 2, 737–751. 10.3390/nu2070737 22254051 PMC3257683

[B129] Mohammadi A. Blesso C. N. Barreto G. E. Banach M. Majeed M. Sahebkar A. (2019). Macrophage Plasticity, Polarization and Function in Response to Curcumin, a Diet-Derived Polyphenol, as an Immunomodulatory Agent. J. Nutr. Biochem. 66, 1–16. 10.1016/j.jnutbio.2018.12.005 30660832

[B130] Monteiro J. P. Bennett M. Rodor J. Caudrillier A. Ulitsky I. Baker A. H. (2019). Endothelial Function and Dysfunction in the Cardiovascular System: the Long Non-coding Road. Cardiovasc. Res. 115, 1692–1704. 10.1093/cvr/cvz154 31214683 PMC6755355

[B131] Morimoto T. Sunagawa Y. Kawamura T. Takaya T. Wada H. Nagasawa A. (2008). The Dietary Compound Curcumin Inhibits P300 Histone Acetyltransferase Activity and Prevents Heart Failure in Rats. J. Clin. Invest. 118, 868–878. 10.1172/JCI33160 18292809 PMC2248328

[B132] Muiesan M. L. Salvetti M. Rosei C. A. Paini A. (2016). Gender Differences in Antihypertensive Treatment: Myths or Legends? High Blood Press. Cardiovasc. Prev. 23, 105–113. 10.1007/s40292-016-0148-1 27106810

[B133] Nakmareong S. Kukongviriyapan U. Pakdeechote P. Donpunha W. Kukongviriyapan V. Kongyingyoes B. (2011). Antioxidant and Vascular Protective Effects of Curcumin and Tetrahydrocurcumin in Rats with L-NAME-Induced Hypertension. Naunyn-schmiedeberg's Arch. Pharmacol. 383, 519–529. 10.1007/s00210-011-0624-z 21448566

[B134] Nakmareong S. Kukongviriyapan U. Pakdeechote P. Kukongviriyapan V. Kongyingyoes B. Donpunha W. (2012). Tetrahydrocurcumin Alleviates Hypertension, Aortic Stiffening and Oxidative Stress in Rats with Nitric Oxide Deficiency. Hypertens. Res. 35, 418–425. 10.1038/hr.2011.180 22072109

[B135] Nelson K. M. Dahlin J. L. Bisson J. Graham J. Pauli G. F. Walters M. A. (2017). The Essential Medicinal Chemistry of Curcumin. J. Med. Chem. 60, 1620–1637. 10.1021/acs.jmedchem.6b00975 28074653 PMC5346970

[B136] Nelson M. T. Patlak J. B. Worley J. F. Standen N. B. (1990). Calcium Channels, Potassium Channels, and Voltage Dependence of Arterial Smooth Muscle Tone. Am. J. Physiology-Cell Physiol. 259, C3–C18. 10.1152/ajpcell.1990.259.1.c3 2164782

[B137] Nguyen K. T. Shaikh N. Shukla K. P. Su S.-H. Eberhart R. C. Tang L. (2004). Molecular Responses of Vascular Smooth Muscle Cells and Phagocytes to Curcumin-Eluting Bioresorbable Stent Materials. Biomaterials 25, 5333–5346. 10.1016/j.biomaterials.2003.12.033 15130718

[B138] Ota K. Kimura T. Shoji M. Ota M. Funyu T. Mori T. (1998). Effects of Endothelin-Induced Nitric Oxide on Venous Circulation and Renal Water-Electrolyte Handling. J. Cardiovasc. Pharmacol. 31 (Suppl. 1), S128–S132. 10.1097/00005344-199800001-00039 9595420

[B139] Ouarné M. Pena A. Franco C. A. (2021). From Remodeling to Quiescence: The Transformation of the Vascular Network. Cells Dev 203735. 10.1016/j.cdev.2021.203735 34425253

[B140] Pae H.-O. Jeong G.-S. Jeong S.-O. Kim H. S. Kim S.-A. Kim Y.-C. (2007). Roles of Heme Oxygenase-1 in Curcumin-Induced Growth Inhibition in Rat Smooth Muscle Cells. Exp. Mol. Med. 39, 267–277. 10.1038/emm.2007.30 17603281

[B141] Pang X. F. Zhang L. H. Bai F. Wang N. P. Garner R. E. Mckallip R. J. (2015). Attenuation of Myocardial Fibrosis with Curcumin Is Mediated by Modulating Expression of Angiotensin II AT1/AT2 Receptors and ACE2 in Rats. Drug Des. Devel Ther. 9, 6043–6054. 10.2147/DDDT.S95333 PMC4651552 26648693

[B142] Panthiya L. Tocharus J. Onsa-Ard A. Chaichompoo W. Suksamrarn A. Tocharus C. (2022). Hexahydrocurcumin Ameliorates Hypertensive and Vascular Remodeling in L-NAME-Induced Rats. Biochim. Biophys. Acta (Bba) - Mol. Basis Dis. 1868, 166317. 10.1016/j.bbadis.2021.166317 34883248

[B143] Paradiso-Hardy F. L. Gordon W. L. Jackevicius C. A. Kertland H. R. Pearson G. J. Pickering J. L. (2003). The Importance of In-Hospital Statin Therapy for Patients with Acute Coronary Syndromes. Pharmacotherapy 23, 506–513. 10.1592/phco.23.4.506.32129 12680480

[B144] Park C.-B. Ahn C. M. Oh S. Kwon D. Cho W.-C. Shin W.-S. (2015). Synthesis of Alkylsulfonyl and Substituted Benzenesulfonyl Curcumin Mimics as Dual Antagonist of L-type Ca2+ Channel and Endothelin A/B2 Receptor. Bioorg. Med. Chem. 23, 6673–6682. 10.1016/j.bmc.2015.09.004 26386817

[B145] Patel S. S. Acharya A. Ray R. S. Agrawal R. Raghuwanshi R. Jain P. (2020). Cellular and Molecular Mechanisms of Curcumin in Prevention and Treatment of Disease. Crit. Rev. Food Sci. Nutr. 60, 887–939. 10.1080/10408398.2018.1552244 30632782

[B146] Pathak N. Khandelwal S. (2008). Comparative Efficacy of Piperine, Curcumin and Picroliv against Cd Immunotoxicity in Mice. Biometals 21, 649–661. 10.1007/s10534-008-9150-y 18566892

[B147] Pechanova O. Dayar E. Cebova M. (2020). Therapeutic Potential of Polyphenols-Loaded Polymeric Nanoparticles in Cardiovascular System. Molecules 25, 3322. 10.3390/molecules25153322 PMC7435870 32707934

[B148] Perros F. Dorfmüller P. Humbert M. (2005). Current Insights on the Pathogenesis of Pulmonary Arterial Hypertension. Semin. Respir. Crit. Care Med. 26, 355–364. 10.1055/s-2005-916149 16121311

[B149] Pinheiro Júnior J. E. G. Moraes P. Z. Rodriguez M. D. Simões M. R. Cibin F. Pinton S. (2020). Cadmium Exposure Activates NADPH Oxidase, Renin-Angiotensin System and Cyclooxygenase 2 Pathways in Arteries, Inducing Hypertension and Vascular Damage. Toxicol. Lett. 333, 80–89. 32738273 10.1016/j.toxlet.2020.07.027

[B150] Prado A. F. Batista R. I. M. Tanus-Santos J. E. Gerlach R. F. (2021). Matrix Metalloproteinases and Arterial Hypertension: Role of Oxidative Stress and Nitric Oxide in Vascular Functional and Structural Alterations. Biomolecules 11, 585. 10.3390/biom11040585 33923477 PMC8074048

[B151] Prieto M. C. Gonzalez A. A. Visniauskas B. Navar L. G. (2021). The Evolving Complexity of the Collecting Duct Renin-Angiotensin System in Hypertension. Nat. Rev. Nephrol. 17, 481–492. 10.1038/s41581-021-00414-6 33824491 PMC8443079

[B152] Pugliese S. C. Poth J. M. Fini M. A. Olschewski A. El Kasmi K. C. Stenmark K. R. (2015). The Role of Inflammation in Hypoxic Pulmonary Hypertension: from Cellular Mechanisms to Clinical Phenotypes. Am. J. Physiology-Lung Cell Mol. Physiol. 308, L229–L252. 10.1152/ajplung.00238.2014 PMC4338929 25416383

[B153] Rabinovitch M. Guignabert C. Humbert M. Nicolls M. R. (2014). Inflammation and Immunity in the Pathogenesis of Pulmonary Arterial Hypertension. Circ. Res. 115, 165–175. 10.1161/circresaha.113.301141 24951765 PMC4097142

[B154] Rachmawati H. Soraya I. S. Kurniati N. F. Rahma A. (2016). *In Vitro* Study on Antihypertensive and Antihypercholesterolemic Effects of Curcumin Nanoemulsion. Sci. Pharm. 84, 131–140. 10.3797/scipharm.isp.2015.05 27110504 PMC4839556

[B155] Rakotoarisoa M. Angelova A. (2018). Amphiphilic Nanocarrier Systems for Curcumin Delivery in Neurodegenerative Disorders. Medicines 5, 126. 10.3390/medicines5040126 PMC6313553 30477087

[B156] Ramirez L. A. Sullivan J. C. (2018). Sex Differences in Hypertension: Where We Have Been and where We Are Going. Am. J. Hypertens. 31, 1247–1254. 10.1093/ajh/hpy148 30299518 PMC6233684

[B157] Rapsomaniki E. Timmis A. George J. Pujades-Rodriguez M. Shah A. D. Denaxas S. (2014). Blood Pressure and Incidence of Twelve Cardiovascular Diseases: Lifetime Risks, Healthy Life-Years Lost, and Age-specific Associations in 1·25 Million People. The Lancet 383, 1899–1911. 10.1016/s0140-6736(14)60685-1 PMC4042017 24881994

[B158] Ren X. S. Tong Y. Qiu Y. Ye C. Wu N. Xiong X. Q. (2020). MiR155‐5p in Adventitial Fibroblasts‐derived Extracellular Vesicles Inhibits Vascular Smooth Muscle Cell Proliferation via Suppressing Angiotensin‐converting Enzyme Expression. J. Extracellular Vesicles 9, 1698795. 10.1080/20013078.2019.1698795 31839907 PMC6896498

[B159] Restini C. B. A. Ismail A. Kumar R. K. Burnett R. Garver H. Fink G. D. (2018). Renal Perivascular Adipose Tissue: Form and Function. Vasc. Pharmacol. 106, 37–45. 10.1016/j.vph.2018.02.004 PMC5990437 29454047

[B160] Rice K. M. Manne N. D. P. K. Kolli M. B. Wehner P. S. Dornon L. Arvapalli R. (2016). Curcumin Nanoparticles Attenuate Cardiac Remodeling Due to Pulmonary Arterial Hypertension. Artif. Cell Nanomedicine, Biotechnol. 44, 1909–1916. 10.3109/21691401.2015.1111235 26631548

[B161] Rizzoni D. Porteri E. Guefi D. Piccoli A. Castellano M. Pasini G. (2000). Cellular Hypertrophy in Subcutaneous Small Arteries of Patients with Renovascular Hypertension. Hypertension 35, 931–935. 10.1161/01.hyp.35.4.931 10775564

[B162] Sakurai T. Yanagisawa M. Takuwat Y. Miyazakit H. Kimura S. Goto K. (1990). Cloning of a cDNA Encoding a Non-isopeptide-selective Subtype of the Endothelin Receptor. Nature 348, 732–735. 10.1038/348732a0 2175397

[B163] Salehi B. Del Prado-Audelo M. L. Cortés H. Leyva-Gómez G. Stojanović-Radić Z. Singh Y. D. (2020). Therapeutic Applications of Curcumin Nanomedicine Formulations in Cardiovascular Diseases. Jcm 9, 746. 10.3390/jcm9030746 PMC7141226 32164244

[B164] Sangartit W. Kukongviriyapan U. Donpunha W. Pakdeechote P. Kukongviriyapan V. Surawattanawan P. (2014). Tetrahydrocurcumin Protects against Cadmium-Induced Hypertension, Raised Arterial Stiffness and Vascular Remodeling in Mice. PLoS One 9, e114908. 10.1371/journal.pone.0114908 25502771 PMC4263715

[B165] Sangartit W. Pakdeechote P. Kukongviriyapan V. Donpunha W. Shibahara S. Kukongviriyapan U. (2016). Tetrahydrocurcumin in Combination with Deferiprone Attenuates Hypertension, Vascular Dysfunction, Baroreflex Dysfunction, and Oxidative Stress in Iron-Overloaded Mice. Vasc. Pharmacol. 87, 199–208. 10.1016/j.vph.2016.10.001 27713040

[B166] Santos-Parker J. R. Strahler T. R. Bassett C. J. Bispham N. Z. Chonchol M. B. Seals D. R. (2017). Curcumin Supplementation Improves Vascular Endothelial Function in Healthy Middle-Aged and Older Adults by Increasing Nitric Oxide Bioavailability and Reducing Oxidative Stress. Aging 9, 187–208. 10.18632/aging.101149 28070018 PMC5310664

[B167] Schiffrin E. L. Deng L. Y. Larochelle P. (1993). Morphology of Resistance Arteries and Comparison of Effects of Vasoconstrictors in Mild Essential Hypertensive Patients. Clin. Invest. Med. 16, 177–186. 8365045

[B168] Selvendiran K. Kuppusamy M. L. Bratasz A. Tong L. Rivera B. K. Rink C. (2009). Inhibition of Vascular Smooth-Muscle Cell Proliferation and Arterial Restenosis by HO-3867, a Novel Synthetic Curcuminoid, through Up-Regulation of PTEN Expression. J. Pharmacol. Exp. Ther. 329, 959–966. 10.1124/jpet.108.150367 19276401

[B169] Shahani K. Swaminathan S. K. Freeman D. Blum A. Ma L. Panyam J. (2010). Injectable Sustained Release Microparticles of Curcumin: a New Concept for Cancer Chemoprevention. Cancer Res. 70, 4443–4452. 10.1158/0008-5472.can-09-4362 20460537 PMC2880212

[B170] Sharma R. A. Euden S. A. Platton S. L. Cooke D. N. Shafayat A. Hewitt H. R. (2004). Phase I Clinical Trial of Oral Curcumin. Clin. Cancer Res. 10, 6847–6854. 10.1158/1078-0432.ccr-04-0744 15501961

[B171] Sharma R. K. Oliveira A. C. Yang T. Karas M. M. Li J. Lobaton G. O. (2020). Gut Pathology and its Rescue by ACE2 (Angiotensin-Converting Enzyme 2) in Hypoxia-Induced Pulmonary Hypertension. Hypertension 76, 206–216. 10.1161/hypertensionaha.120.14931 32418496 PMC7505091

[B172] Sheu M.-J. Lin H.-Y. Yang Y.-H. Chou C.-J. Chien Y.-C. Wu T.-S. (2013). Demethoxycurcumin, a Major Active Curcuminoid from Curcuma Longa , Suppresses Balloon Injury Induced Vascular Smooth Muscle Cell Migration and Neointima Formation: An *In Vitro* and *In Vivo* Study. Mol. Nutr. Food Res. 57, 1586–1597. 10.1002/mnfr.201200462 23520190

[B173] Shi N. Chen S. Y. (2014). Mechanisms Simultaneously Regulate Smooth Muscle Proliferation and Differentiation. J. Biomed. Res. 28, 40–46. 10.7555/JBR.28.20130130 24474962 PMC3904173

[B174] Shishodia S. Sethi G. Aggarwal B. B. (2005). Curcumin: Getting Back to the Roots. Ann. New York Acad. Sci. 1056, 206–217. 10.1196/annals.1352.010 16387689

[B175] Shome S. Talukdar A. D. Choudhury M. D. Bhattacharya M. K. Upadhyaya H. (2016). Curcumin as Potential Therapeutic Natural Product: a Nanobiotechnological Perspective. J. Pharm. Pharmacol. 68, 1481–1500. 10.1111/jphp.12611 27747859

[B176] Soni K. B. Kuttan R. (1992). Effect of Oral Curcumin Administration on Serum Peroxides and Cholesterol Levels in Human Volunteers. Indian J. Physiol. Pharmacol. 36, 273–275. 1291482

[B177] Sørensen S. S. Jensen J. D. Madsen J. K. Pedersen E. B. (1995). Effect of Isradipine on Renal Haemodynamics and Systemic Blood Pressure Changes Induced by Intravenous Infusion of Endothelin in Healthy Humans. Nephrol. Dial. Transpl. 10, 1324–1331. 8538922

[B178] Srinivasan M. (1972). Effect of Curcumin on Blood Sugar as Seen in a Diabetic Subject. Indian J. Med. Sci. 26, 269–270. 4637293

[B179] Stenmark K. R. Tuder R. M. El Kasmi K. C. (20151985). Metabolic Reprogramming and Inflammation Act in Concert to Control Vascular Remodeling in Hypoxic Pulmonary Hypertension. J. Appl. Physiol. 119, 1164–1172. 10.1152/japplphysiol.00283.2015 PMC4816410 25930027

[B180] Stöger J. L. Gijbels M. J. Van Der Velden S. Manca M. Van Der Loos C. M. Biessen E. A. (2012). Distribution of Macrophage Polarization Markers in Human Atherosclerosis. Atherosclerosis 225, 461–468. 10.1016/j.atherosclerosis.2012.09.013 23078881

[B181] Stumpf C. Raaz D. Klinghammer L. Schneider M. Schmieder R. E. Garlichs C. D. (2016). Platelet CD40 Contributes to Enhanced Monocyte Chemoattractant Protein 1 Levels in Patients with Resistant Hypertension. Eur. J. Clin. Invest. 46, 564–571. 10.1111/eci.12635 27090943

[B182] Sun H.-J. Hou B. Wang X. Zhu X.-X. Li K.-X. Qiu L.-Y. (2016). Endothelial Dysfunction and Cardiometabolic Diseases: Role of Long Non-coding RNAs. Life Sci. 167, 6–11. 10.1016/j.lfs.2016.11.005 27838210

[B183] Sun H.-J. Ren X.-S. Xiong X.-Q. Chen Y.-Z. Zhao M.-X. Wang J.-J. (2017a). NLRP3 Inflammasome Activation Contributes to VSMC Phenotypic Transformation and Proliferation in Hypertension. Cell Death Dis 8, e3074. 10.1038/cddis.2017.470 28981106 PMC5680591

[B184] Sun H. J. Wu Z. Y. Nie X. W. Bian J. S. (2019b). Role of Endothelial Dysfunction in Cardiovascular Diseases: The Link between Inflammation and Hydrogen Sulfide. Front. Pharmacol. 10, 1568. 10.3389/fphar.2019.01568 32038245 PMC6985156

[B185] Sun H. J. Zhu X. X. Cai W. W. Qiu L. Y. (2017b). Functional Roles of Exosomes in Cardiovascular Disorders: a Systematic Review. Eur. Rev. Med. Pharmacol. Sci. 21, 5197–5206. 10.26355/eurrev_201711_13840 29228434

[B186] Sun H. J. Wu Z. Y. Cao L. Zhu M. Y. Liu T. T. Guo L. (2019a). Hydrogen Sulfide: Recent Progression and Perspectives for the Treatment of Diabetic Nephropathy. Molecules 24, 2857. 10.3390/molecules24152857 PMC6696501 31390847

[B187] Sun S.-y. Cao Y.-m. Huo Y.-j. Qiu F. Quan W.-j. He C.-p. (2021). Nicotinate-curcumin Inhibits AngII-Induced Vascular Smooth Muscle Cell Phenotype Switching by Upregulating Daxx Expression. Cell Adhes. Migration 15, 116–125. 10.1080/19336918.2021.1909899 PMC8043179 33843453

[B188] Sunagawa Y. Funamoto M. Shimizu K. Shimizu S. Sari N. Katanasaka Y. (2021). Curcumin, an Inhibitor of P300-HAT Activity, Suppresses the Development of Hypertension-Induced Left Ventricular Hypertrophy with Preserved Ejection Fraction in Dahl Rats. Nutrients 13, 2608. 10.3390/nu13082608 34444769 PMC8397934

[B189] Tamás P. Worgall S. Sulyok E. Rascher W. (1994). Renal Electrolyte and Water Handling in normal Pregnancy: Possible Role of Endothelin-1. Eur. J. Obstet. Gynecol. Reprod. Biol. 55, 89–95. 10.1016/0028-2243(94)90060-4 7958155

[B190] Tapia E. Soto V. Ortiz-Vega K. M. Zarco-Márquez G. Molina-Jijón E. Cristóbal-García M. (2012). Curcumin Induces Nrf2 Nuclear Translocation and Prevents Glomerular Hypertension, Hyperfiltration, Oxidant Stress, and the Decrease in Antioxidant Enzymes in 5/6 Nephrectomized Rats. Oxid Med. Cel Longev 2012, 269039. 10.1155/2012/269039 PMC3424005 22919438

[B191] Thenappan T. Ormiston M. L. Ryan J. J. Archer S. L. (2018). Pulmonary Arterial Hypertension: Pathogenesis and Clinical Management. Bmj 360, j5492. 10.1136/bmj.j5492 29540357 PMC6889979

[B192] Tipton A. J. Sullivan J. C. (2014). Sex Differences in T Cells in Hypertension. Clin. Ther. 36, 1882–1900. 10.1016/j.clinthera.2014.07.011 25134971 PMC4267900

[B193] Tong Y. Ye C. Ren X.-S. Qiu Y. Zang Y.-H. Xiong X.-Q. (2018). Exosome-Mediated Transfer of ACE (Angiotensin-Converting Enzyme) from Adventitial Fibroblasts of Spontaneously Hypertensive Rats Promotes Vascular Smooth Muscle Cell Migration. Hypertension 72, 881–888. 10.1161/hypertensionaha.118.11375 30354715

[B194] Tong Y. Ye C. Zheng F. Bo J.-H. Wu L.-L. Han Y. (2021). Extracellular Vesicle-Mediated miR135a-5p Transfer in Hypertensive Rat Contributes to Vascular Smooth Muscle Cell Proliferation via Targeting FNDC5. Vasc. Pharmacol. 140, 106864. 10.1016/j.vph.2021.106864 33865997

[B195] Tønnesen H. H. Másson M. Loftsson T. (2002). Studies of Curcumin and Curcuminoids. XXVII. Cyclodextrin Complexation: Solubility, Chemical and Photochemical Stability. Int. J. Pharm. 244, 127–135. 12204572 10.1016/s0378-5173(02)00323-x

[B196] Toral M. Robles‐Vera I. Visitación N. Romero M. Sánchez M. Gómez‐Guzmán M. (2019). Role of the Immune System in Vascular Function and Blood Pressure Control Induced by Faecal Microbiota Transplantation in Rats. Acta Physiol. 227, e13285. 10.1111/apha.13285 31004464

[B197] Tubsakul A. Sangartit W. Pakdeechote P. Kukongviriyapan V. Apaijit K. Kukongviriyapan U. (2021). Curcumin Mitigates Hypertension, Endothelial Dysfunction and Oxidative Stress in Rats with Chronic Exposure to Lead and Cadmium. Tohoku J. Exp. Med. 253, 69–76. 10.1620/tjem.253.69 33473064

[B198] Tuder R. M. (2017). Pulmonary Vascular Remodeling in Pulmonary Hypertension. Cell Tissue Res 367, 643–649. 10.1007/s00441-016-2539-y 28025704 PMC5408737

[B199] Tuder R. M. Stacher E. Robinson J. Kumar R. Graham B. B. (2013). Pathology of Pulmonary Hypertension. Clin. Chest Med. 34, 639–650. 10.1016/j.ccm.2013.08.009 24267295

[B200] Usharani P. Mateen A. A. Naidu M. U. R. Raju Y. S. N. Chandra N. (2008). Effect of NCB-02, Atorvastatin and Placebo on Endothelial Function, Oxidative Stress and Inflammatory Markers in Patients with Type 2 Diabetes Mellitus. Drugs R. D 9, 243–250. 10.2165/00126839-200809040-00004 18588355

[B201] Van Beusecum J. P. Moreno H. Harrison D. G. (2021). Innate Immunity and Clinical Hypertension. J. Hum. Hypertens. 10.1038/s41371-021-00627-z PMC9125746 34689174

[B202] Virdis A. Taddei S. (2016). Endothelial Dysfunction in Resistance Arteries of Hypertensive Humans. J. Cardiovasc. Pharmacol. 67, 451–457. 10.1097/fjc.0000000000000362 26808712

[B203] Wang B. Sheng Y. Li Y. (2021a). Lymphatic Microcirculation Profile in the Progression of Hypertension in Spontaneously Hypertensive Rats. Microcirculation, e12724. 10.1111/micc.12724 34351675 PMC9787898

[B204] Wang G. Zhu Y. Li K. Liao B. Wang F. Shao L. (2021b). Curcumin-mediated Photodynamic Therapy Inhibits the Phenotypic Transformation, Migration, and Foaming of Oxidized Low-Density Lipoprotein-Treated Vascular Smooth Muscle Cells by Promoting Autophagy. J. Cardiovasc. Pharmacol. 78, 308–318. 10.1097/fjc.0000000000001069 34091481 PMC8340951

[B205] Wang H. X. Davis M. J. Rajanayagam M. A. S. Potocnik S. J. Hill M. A. (1999). Myogenic Reactivity of Rat Epineurial Arterioles: Potential Role in Local Vasoregulatory Events. Am. J. Physiology-Heart Circulatory Physiol. 277, H144–H151. 10.1152/ajpheart.1999.277.1.h144 10409192

[B206] Webb M. L. Meek T. D. (1997). Inhibitors of Endothelin. Med. Res. Rev. 17, 17–67. 10.1002/(sici)1098-1128(199701)17:1<17::aid-med2>3.0.co;2-w 8979248

[B207] Wickenberg J. Ingemansson S. L. Hlebowicz J. (2010). Effects of Curcuma Longa (Turmeric) on Postprandial Plasma Glucose and Insulin in Healthy Subjects. Nutr. J. 9, 43. 10.1186/1475-2891-9-43 20937162 PMC2964546

[B208] Wu N. Ye C. Zheng F. Wan G.-W. Wu L.-L. Chen Q. (2020). MiR155-5p Inhibits Cell Migration and Oxidative Stress in Vascular Smooth Muscle Cells of Spontaneously Hypertensive Rats. Antioxidants 9, 204. 10.3390/antiox9030204 PMC7140008 32121598

[B209] Xiang D. Li Y. Cao Y. Huang Y. Zhou L. Lin X. (2021). Different Effects of Endothelial Extracellular Vesicles and LPS-Induced Endothelial Extracellular Vesicles on Vascular Smooth Muscle Cells: Role of Curcumin and its Derivatives. Front. Cardiovasc. Med. 8, 649352. 10.3389/fcvm.2021.649352 34150863 PMC8210670

[B210] Xiao Q. Li X. Li Y. Wu Z. Xu C. Chen Z. (2021). Biological Drug and Drug Delivery-Mediated Immunotherapy. Acta Pharmaceutica Sinica B 11, 941–960. 10.1016/j.apsb.2020.12.018 33996408 PMC8105778

[B211] Xu S. Ilyas I. Little P. J. Li H. Kamato D. Zheng X. (2021). Endothelial Dysfunction in Atherosclerotic Cardiovascular Diseases and beyond: From Mechanism to Pharmacotherapies. Pharmacol. Rev. 73, 924–967. 10.1124/pharmrev.120.000096 34088867

[B212] Xu X.-Y. Meng X. Li S. Gan R.-Y. Li Y. Li H.-B. (2018). Bioactivity, Health Benefits, and Related Molecular Mechanisms of Curcumin: Current Progress, Challenges, and Perspectives. Nutrients 10, 1553. 10.3390/nu10101553 PMC6213156 30347782

[B213] Yallapu M. M. Jaggi M. Chauhan S. C. (2012). Curcumin Nanoformulations: a Future Nanomedicine for Cancer. Drug Discov. Today 17, 71–80. 10.1016/j.drudis.2011.09.009 21959306 PMC3259195

[B214] Yallapu M. M. Nagesh P. K. B. Jaggi M. Chauhan S. C. (2015). Therapeutic Applications of Curcumin Nanoformulations. Aaps j 17, 1341–1356. 10.1208/s12248-015-9811-z 26335307 PMC4627456

[B215] Yamamoto T. Kimura T. Ota K. Shoji M. Inoue M. Sato K. (1991). Central Effects of Endothelin-1 on Vasopressin and Atrial Natriuretic Peptide Release and Cardiovascular and Renal Function in Conscious Rats. J. Cardiovasc. Pharmacol. 17 (Suppl. 7), S316–S318. 10.1097/00005344-199100177-00090 1725367

[B216] Yang X. Thomas D. P. Zhang X. Culver B. W. Alexander B. M. Murdoch W. J. (2006). Curcumin Inhibits Platelet-Derived Growth Factor-Stimulated Vascular Smooth Muscle Cell Function and Injury-Induced Neointima Formation. Atvb 26, 85–90. 10.1161/01.atv.0000191635.00744.b6 16239599

[B217] Yao Y. Wang W. Li M. Ren H. Chen C. Wang J. (2016). Curcumin Exerts its Anti-hypertensive Effect by Down-Regulating the AT1 Receptor in Vascular Smooth Muscle Cells. Sci. Rep. 6, 25579. 10.1038/srep25579 27146402 PMC4857140

[B218] Yaribeygi H. Maleki M. Majeed M. Jamialahmadi T. Sahebkar A. (2021). Renoprotective Roles of Curcumin. Adv. Exp. Med. Biol. 1328, 531–544. 10.1007/978-3-030-73234-9_38 34981504

[B219] Young N. A. Bruss M. S. Gardner M. Willis W. L. Mo X. Valiente G. R. (2014). Oral Administration of Nano-Emulsion Curcumin in Mice Suppresses Inflammatory-Induced NFκB Signaling and Macrophage Migration. PLoS One 9, e111559. 10.1371/journal.pone.0111559 25369140 PMC4219720

[B220] Yu Y.-M. Lin H.-C. (2010). Curcumin Prevents Human Aortic Smooth Muscle Cells Migration by Inhibiting of MMP-9 Expression. Nutr. Metab. Cardiovasc. Dis. 20, 125–132. 10.1016/j.numecd.2009.03.001 19447587

[B221] Zeng X. Cheng Y. Qu Y. Xu J. Han Z. Zhang T. (2013). Curcumin Inhibits the Proliferation of Airway Smooth Muscle Cells *In Vitro* and *In Vivo* . Int. J. Mol. Med. 32, 629–636. 10.3892/ijmm.2013.1425 23807697

[B222] Zhang H.-n. Xu Q.-q. Thakur A. Alfred M. O. Chakraborty M. Ghosh A. (2018). Endothelial Dysfunction in Diabetes and Hypertension: Role of microRNAs and Long Non-coding RNAs. Life Sci. 213, 258–268. 10.1016/j.lfs.2018.10.028 30342074

[B223] Zhang J.-R. Sun H.-J. (2022). Extracellular Vesicle-Mediated Vascular Cell Communications in Hypertension: Mechanism Insights and Therapeutic Potential of ncRNAs. Cardiovasc. Drugs Ther. 36, 157–172. 10.1007/s10557-020-07080-z 32964302

[B224] Zhang J.-R. Sun H.-J. (2020). LncRNAs and Circular RNAs as Endothelial Cell Messengers in Hypertension: Mechanism Insights and Therapeutic Potential. Mol. Biol. Rep. 47, 5535–5547. 10.1007/s11033-020-05601-5 32567025

[B225] Zhang J.-R. Sun H.-J. (2021). MiRNAs, lncRNAs, and Circular RNAs as Mediators in Hypertension-Related Vascular Smooth Muscle Cell Dysfunction. Hypertens. Res. 44, 129–146. 10.1038/s41440-020-00553-6 32985618

[B226] Zhang M. Li Y. Xie H. Chen J. Liu S. (2020). Curcumin Inhibits Proliferation, Migration and Neointimal Formation of Vascular Smooth Muscle via Activating miR-22. Pharm. Biol. 58, 610–619. 10.1080/13880209.2020.1781904 32631202 PMC8641690

[B227] Zheng Y. Xu Z. (2014). MicroRNA-22 Induces Endothelial Progenitor Cell Senescence by Targeting AKT3. Cell Physiol Biochem 34, 1547–1555. 10.1159/000366358 25323119

[B228] Zhou Y. Zhang T. Wang X. Wei X. Chen Y. Guo L. (2015). Curcumin Modulates Macrophage Polarization through the Inhibition of the Toll-like Receptor 4 Expression and its Signaling Pathways. Cel Physiol Biochem 36, 631–641. 10.1159/000430126 25998190

[B229] Zhu X. Zhou Z. Zhang Q. Cai W. Zhou Y. Sun H. (2018). Vaccarin Administration Ameliorates Hypertension and Cardiovascular Remodeling in Renovascular Hypertensive Rats. J. Cel. Biochem. 119, 926–937. 10.1002/jcb.26258 28681939

